# Nematode and Arthropod Genomes Provide New Insights into the Evolution of Class 2 B1 GPCRs

**DOI:** 10.1371/journal.pone.0092220

**Published:** 2014-03-20

**Authors:** João C. R. Cardoso, Rute C. Félix, Deborah M. Power

**Affiliations:** Comparative Molecular and Integrative Biology, Centre of Marine Sciences, Faro, Portugal; Wake Forest University, United States of America

## Abstract

Nematodes and arthropods are the most speciose animal groups and possess Class 2 B1 G-protein coupled receptors (GPCRs). Existing models of invertebrate Class 2 B1 GPCR evolution are mainly centered on *Caenorhabditis elegans* and *Drosophila melanogaster* and a few other nematode and arthropod representatives. The present study reevaluates the evolution of metazoan Class 2 B1 GPCRs and orthologues by exploring the receptors in several nematode and arthropod genomes and comparing them to the human receptors. Three novel receptor phylogenetic clusters were identified and designated cluster A, cluster B and PDF-R-related cluster. Clusters A and B were identified in several nematode and arthropod genomes but were absent from *D. melanogaster* and Culicidae genomes, whereas the majority of the members of the PDF-R-related cluster were from nematodes. Cluster A receptors were nematode and arthropod-specific but shared a conserved gene environment with human receptor loci. Cluster B members were orthologous to human GCGR, PTHR and Secretin members with which they probably shared a common origin. PDF-R and PDF-R related clusters were present in representatives of both nematodes and arthropods. The results of comparative analysis of GPCR evolution and diversity in protostomes confirm previous notions that *C. elegans* and *D. melanogaster* genomes are not good representatives of nematode and arthropod phyla. We hypothesize that at least four ancestral Class 2 B1 genes emerged early in the metazoan radiation, which after the protostome-deuterostome split underwent distinct selective pressures that resulted in duplication and deletion events that originated the current Class 2 B1 GPCRs in nematode and arthropod genomes.

## Introduction

In tetrapods, five main G protein-coupled receptor (GPCR) gene families have been identified and are characterized by the presence of seven helical transmembrane (TM) structures [1], [2]. The Secretin-like family of GPCRs, named after the receptor that binds to secretin, the first hormone identified in vertebrates [3]–[5], is also known as Class 2 (II or B) subfamily (or subclass) B1 GPCRs (Class 2 B1) and their members are only activated by polypeptide hormones [6]–[8]. In humans there are 15 peptide-binding receptors and they are involved in the regulation of a wide spectrum of endocrine and neuroendocrine functions including cell growth, development, calcium homeostasis, stress response, immune function and brain-gut functions including muscle motility, feeding regulation and glucose metabolism [9]–[12] (Table 1). Class 2 B1 share low sequence and structure homology with other GPCR families but are highly conserved when basal vertebrates such as lamprey and hagfish are compared with mammals including humans [13]–[16]. Unique features of Class 2 B1 GPCRs are the presence of large N-terminal ligand-binding ectodomain (N-ted) that contain six conserved cysteines and several N-glycosylation motifs [17]–[19]. Their ligands are moderately large peptides that are members of distinct endocrine peptide families and the vertebrate receptors have been grouped into five subfamilies and include the receptors for: a) calcitonin (CALC) and calcitonin gene related peptide (CGRP), b) corticotropin hormone (CRH), c) parathyroid and related peptides (PTH and PTHrP), d) glucagon (GCG) and related peptides (GLP) and e) secretin (SCT), vasoactive intestinal peptide (VIP), pituitary adenylate cyclase activating polypeptide (PACAP) and growth hormone releasing hormone (GHRH). Receptor activation occurs when peptides interact with the N-terminal domain and TM loops and this triggers intracellular signaling via adenylate cyclase and/or an increase in calcium ions.

**Table 1:** Physiological role of Class 2 B1 members in the nematode *C. elegans*, fruit-fly *D. melanogaster* and human.

	Receptors	Ligands	Physiological roles	References
***C. elegans***				
	**PDF-R (a,b,c)**	PDF-1a,b, 2	Circadian rhythms, locomotion,reproduction, gastrointestinal regulation^b^	[26], [67], [68]
	**Seb-2 (a** a **,b)**	*n.i.*	Head movement^b^, Vulva contraction^b^,	
	**Seb-3**	*n.i.*	Locomotion, stress response, ethanoltolerance, neuronal regulation^b^	[67], [69]
***D. melanogaster***				
	**PDF-R**	PDF	Circadian and geotactic rhythms, visceralphysiology, reproduction, activity, arousal	[53], [70]–[72]
	**DH31-R**	DH31	Water excretion, diuresis,digestive functions^c^	[55], [60], [61], [72]
	**DH44-R1,** **DH44-R2**	DH44	Water excretion, osmose balance,diuresis, digestive functions^c^	[21], [22], [57], [59], [72]
				
	**HecR**	*n.i.*	Male courtship behavior	[29]
**Human**				
Calcitoninreceptors	**CALCR,** **CALCRL**	CALC, IAPP^d^,CGRP, ADM^d^	Vascular relaxation and vasodilatation,calcium and phosphorous metabolism	[73], [74]
Corticotropinreceptors	**CRHR1,** **CRHR2**	CRH, UCN,UCN II, UCN III	ACTH secretion, stress response, food intake,satiety, homeostatic balance, vascular tone	[75]–[77]
Parathyroid andrelated peptidereceptors	**PTH1R,** **PTH2R**	PTH, PTHrP,TIP39	Calcium and phosphorous metabolism, bonedevelopment, stress response, growthhormone secretion, arginine-vasopressinrelease	[78], [79]
Glucagon and relatedpeptide receptors	**GLP1R, GLP2R,** **GIPR, GCGR**	GCG, GLP-1,GLP-2, GIP	Insulin pancreatic secretion; fatty acidmetabolism, satiety, gluconeogenesis, glycogenolysis, intestinal growth	[80]–[82]
Secretinreceptors	**SCTR, VIPR1, VIPR2,** **ADCYAP1R, GHRHR**	SCT, VIP,PACAP, GHRH	Pancreatic secretion, inhibition of gastric acidsecretion, neuromodulation, neuroprotection,T-cell differentiation, circadian rhythms,pituitary hormone release	[12], [83]–[86]

The human receptor gene symbols are in agreement with the IUPHAR database (www.iuphar-db.org) pdf, Pigment-dispersing factor; pdf-r, pdf receptor; Seb, Secretin/Class B GPCRs; DH31, Diuretic hormone 31; DH31-R, DH31 receptor; DH44, Diuretic hormone 44; DH44-R, DH44 receptor; Hec-R, hector receptor; CALC, Calcitonin; CALCR, CALC receptor; CALCRL, CALC-like receptor; IAPP, Amylin; CGRP, CALC gene related peptide; ADM, Adrenomedullin; CRH, Corticotropin-releasing hormone; CRHR, CRH receptor; UCT, Urocortin; PTH, Parathyroid hormone; PTHR, PTH receptor; PTHrP, PTH-related peptide; TIP39, Tuberoinfundibular peptide of 39 residues; GLP, Glucagon-like peptide, GLPR, GLP receptor; GCG, Glucagon; GCGR, GCG receptor; GIP, gastric inhibitory polypeptide; GIPR, GIP receptor; SCT, Secretin; SCTR, secretin receptor; VIP, Vasoactive intestinal peptide; VIPR1, VIP receptor 1 (VPAC1); VIPR2, VIP receptor 2 (VPAC2); PACAP, Pituitary adenylate cyclase activating polypeptide; ADCYAP1R, PACAP receptor (PAC1); GHRH, Growth hormone releasing hormone; GHRHR, GHRH receptor.

a only six TM regions predicted,

b Predicted function based on expression data available from Wormbase (www.wormbase.org);

c Predicted function based on expression pattern obtained from [72];

d Receptor activation via interactions with accessory proteins RAMP1, RAMP2 and RAMP3; *n.i.* not identified.

Homologues of the vertebrate GPCRs have also been identified in invertebrates and Class 2 B1 members were suggested to descend from the family of Adhesion GPCRs, a more ancient GPCR family with representatives in early eukaryotes [20]. In the genomes of the nematode *Caenorhabditis elegans* 3 Class 2 B1 receptors have been predicted and in the fruit fly *Drosophila melanogaster* 5 receptors have been described and they are related in sequence and function with the vertebrate CALCR and CRHRs [13], [21], [22]. In other invertebrates, receptor homologues have also been characterized but the majority has no known function and remain orphans [23]–[25]. The few receptors with established functions are involved in the regulation of ion transport, locomotion, circadian rhythm and behavior and include *D. melanogaster* diuretic hormone (DH) receptors: DH31-R and duplicate DH44-R (DH44-R1 and DH44-R2); the *D. melanogaster* orphan receptor, Hector (Hec) and the *D. melanogaster* and *C. elegans* pigment-dispersing factor (PDF) receptor (PDF-R) (Table 1) [22], [26]–[29]. At present the function of two *C. elegans* orphan receptors Secretin/class B GPCR (Seb-2 and Seb-3) are poorly understood.

Nematodes and arthropods are two of the most diverse animal phyla but most phylogenetic analysis has focused on the genomes of the model species *C. elegans* and *D.* melanogaster. Recently, two studies characterizing the insect and bilaterian Class 2 B1 GPCR evolution suggested that in insect genomes excluding *D. melanogaster*, a group of receptors that are related to the vertebrate PTHR, GCG and Secretin subfamilies exists [30], [31]. Moreover, the phylogenetic trees presented [31] suggests the possible existence of novel receptor clusters but analysis with a far greater number of insect and bilaterian representatives are required to resolve this issue. Comparative analysis of genome data from distinct protostomes should contribute to provide more accurate models of metazoan gene evolution and in this context, it was recently demonstrated that gene evolution of human peptide-rhodopsin GPCR orthologues in nematodes and arthropods had taken different paths despite their similar receptor repertoire [32]. In particular, gene number of rhodopsin GPCRs in diverse nematode and arthropod genomes was congruent with species-specific gene duplications and deletions presumably due to their differing life-styles and, for example, the genomes of parasitic nematodes have lost genes compared to free-living forms [32].

In the present study the evolution of metazoan Class 2 B1 GPCRs and orthologues is reevaluated and incorporates in addition to *C. elegans* and *D. melanogaster* receptors those retrieved from other nematode and arthropod genomes and compares them to human. In nematodes, 2 to 4 members were characterized while in arthropods gene number varied from 5 to 11 supporting the notion that receptor gene evolution within and between nematodes and arthropods was distinct. In nematode and arthropod genomes three novel phylogenetic receptor Class B1 clusters were identified and named cluster A, cluster B and PDF-R-related cluster, and no representatives were found in Diptera genomes. Members of cluster A included *C.* elegans Seb-3 and the PDF-R-related cluster contained *C. elegans* Seb-2. Cluster B members grouped with human GCGR, PTHR, and SCTR subfamily members (hereafter designated by GPS-receptor group) indicating that the common ancestral receptor gene was present before the protostome-deuterostome lineage split. Orthologues of the *D. melanogaster* DH44-R, DH31-R and Hec-R were identified only in arthropods and DH31-R and Hec-R are evolutionary closely related and group with the human CALCR. The arthropod DH44-R tended to group with the human CRHR and in the same branch as the nematode and arthropod representatives of PDF-Rs and novel PDF-R related clusters. Receptor gene environment revealed that despite their divergence conserved gene linkage across *C. elegans*, *Tribolium castaneum* and the vertebrates chicken and human exist and data supports the evolutionary models that propose they arose early during the metazoan radiation.

## Materials and Methods

### Database Searches

Sequence database searches using the deduced complete protein sequence of the *C. elegans* and *D. melanogaster* Class 2 B1 members were carried out in nematode and arthropod genomes publicly available. Of the phylum Nematoda, 6 genomes were analyzed, which represented 3 different nematode classes, Chromadorea, Secernentea and Enoplea. The genomes analyzed in Chromadorea included *C. elegans*, *Haemonchus contortus* and *Pristionchus pacificus*; in Secernentea, *Meloidogyne incognita* and *Brugia malayi* and in Enoplea *Trichinella spiralis*. The *H. contortus* sequences were retrieved from the Sanger database (http://www.sanger.ac.uk/), the *M. incognita* sequences were obtained from the INRA database (http://meloidogyne.toulouse.inra.fr) and the sequences of *C. elegans*, *T. spiralis*, *P. pacificus* and *B. malayi* were retrieved from the Wormbase (http://www.wormbase.org) and NCBI (http://blast.ncbi.nlm.nih.gov) databases.

A total of 18 arthropod genomes, which included representatives of the Insecta, the Arachnida and the Branchiopoda classes, were also explored. All the sequences were retrieved from Ensembl Metazoa (http://metazoa.ensembl.org/index.html) and the *D. melanogaster* sequences were also obtained from Flybase (http://www.flybase.org). Members of the insect class included; the Diptera, *D. melanogaster* and representatives of the Culicidae family, *Aedes aegypti*, *Anopheles gambiae*, *Anopheles darlingi* and *Culex quinquefasciatus*; Hymenoptera, *Apis mellifera*, *Nasonia vitripennis* and *Atta cephalotes*; Coleoptera, *Tribolium castaneum*; Lepidoptera, *Bombyx mori*, *Danaus plexippus* and *Heliconius melpomene*; Hemiptera, *Acyrthosiphon pisum* and *Rhodnius prolixus*; and Phthiraptera, *Pediculus humanus*. Species of the Arachnida class included *Ixodes scapularis* and *Tetranychus urticae* and of the Branchiopoda class the *Daphnia pulex* genome. The putative invertebrate Class 2 B1 receptors were extracted from their genomes and sequence identity with *C. elegans* and *D. melanogaster* homologues was confirmed.

Searches of EST data (http://www.ncbi.nlm.nih.gov/dbEST/) was also carried out for the Nematoda (*C. elegans,* 396687 EST; *H. contortus*, 21975 EST; *P. pacificus,* 37470 EST; *M. incognita*, 63838 EST; *B. malayi*, 26215 EST; and *T. spiralis,* 25268 EST) and the Arthropoda (*T. castaneum*, 64571 EST; *D. pulex*, 152659 EST; *A. mellifera*, 169497 EST; *P. humanus*, 4508 EST; *I. scapularis*, 193773 EST; *H. melpomene*, 6004 EST; *B. mori*, 568825 EST; *D. plexippus*, 19577 EST; *Nasonia vitripennis*, 145793 EST; *Rhodnius prolixus* 16105 EST; *Tetranychus urticae*, 80855 EST) to establish, using an *in silico* approach, the putative tissue distribution of cluster A and cluster B receptors. No ESTs for *A. cephalotes* were available.

Searches for putative deuterostome cluster A and PDF-R gene homologues were performed in the chordate genomes: Ciona (*Ciona intestinalis,* www.ensembl.org); amphioxus (*Branchiostoma floridae,* http://genome.jgi-psf.org); sea urchins (*Strongylocentrotus purpuratus,* www.ensemblgenomes.org, *Strongylocentrotus franciscanus* https://www.hgsc.bcm.edu/, *Lytechinus variegatus*, http://www.equinoxbase.com and *Allocentrotus fragilis,* https://www.hgsc.bcm.edu/), sea star (*Patiria miniata,* http://www.equinoxbase.com); and the Hemichordate the acorn worm (*Saccoglossus kowalevskii*, https://www.hgsc.bcm.edu). Searches were also performed in vertebrates (human, chicken and fish) and also in lamprey (*Petromyzus marinus*) available from www.ensembl.org.

### Sequence Comparisons and Alignments

The deduced amino acid sequences of retrieved Class 2 B1 members were compared with the *C. elegans* and *D. melanogaster* receptor homologues and conserved transmembrane regions (TM) were identified using the TMHMM tool (http://www.cbs.dtu.dk/services/TMHMM/) and previous annotations [13]. TM domains were incomplete or were absent from several of the Class 2 B1 genes predicted *in silico*. To obtain the most complete receptor TM core, homology searches were performed and the missing TM or incomplete sequences were directly retrieved from the genome assembly. TM domains were concatenated and used to interrogate the *C. elegans*, *D. melanogaster* and human databases to confirm identity. The nematode and arthropod receptor TM domains were aligned using ClustalW (http://www.genome.jp/tools/clustalw/) and manually edited when TM orthologous relationships were incorrectly predicted (Figure S1). Only unique receptor genes were used in the analysis and they were identified based upon the presence of overlapping TM domains and their distinct genome localizations. To produce receptors with longer TM cores several predictions for the same gene were combined when possible.

Only the TM domains of nematode and arthropod receptors were used to calculate sequence identity/similarity and for phylogenetic tree construction. Percentages of amino acid sequence identity/similarity of TM domains were calculated using GeneDoc software (http://www.nrbsc.org/gfx/genedoc/).

The N-terminal region (N-ted) of full-length nematode (*C. elegans* and *T. spiralis*) and arthropod (*D. melanogaster, A. gambiae* and *T. castaneum*) Class 2 B1 receptors were retrieved and the receptor sequence upstream of TM1 were aligned and compared with human sequence homologues: CHRH2 (ENSG00000106113), CALCR (ENSG00000004948), PTH1R (ENSG00000160801), VPAC_1_ (ENSG00000114812) and GCGR (ENSG00000215644) to search for conserved motifs between the vertebrate and invertebrate receptors and also within specific metazoan families. Comparisons were performed using the most conserved N-terminal region within the species. The position of cysteines, putative consensus N-glycosylation sites (N-X-S/T) and other amino acid motifs characteristic of the members of Class 2 B1 and involved in vertebrate receptor function were annotated.

### Phylogenetic Analysis

Phylogenetic analysis was carried out using the conserved receptor TM domains of the nematode and arthropod putative Class 2 B1 members (Figure S1). Class 2 B1 GPCR TM regions are highly conserved monophyletic receptor protein domains. Trees were constructed using the edited TM sequence alignments and the Neighbor Joining (NJ) [33] and Maximum Likelihood (ML) methods with bootstraps [34] and both methods generated trees with a similar topology. To select the best model for receptor protein evolution the TM alignment was submitted to ProtTest (2.4) analysis according to the Akaike Information Criterion (AIC) and the JTT amino acid substitution model was selected. The NJ analysis was carried out in MEGA5 [35] and ML was implemented in the PhyML program (v3.0 aLRT) using the Phylogeny.fr platform (http://www.phylogeny.fr/) [36].

NJ analysis was performed by aligning TM domains using ClustalW (v2.0.3) [37] and the tree was constructed with 1000 bootstrap replicates, pairwise deletion for gaps/missing data treatment option with uniform rates among sites or fixed gamma 4 distributed rate categories (gamma = 1.12) to account for rate heterogeneity across sites. Both NJ and ML analysis generated similar tree topologies. The evolutionary distances were computed in units of number of amino acid substitutions per site. Ambiguous sequences were removed from the final dataset (total of 121 receptor sequences). In ML analysis sequences were aligned using ClustalW (v2.0.3) [37] and trees constructed using the JTT substitution model assuming an estimated proportion of invariant sites (0.01) and 4 gamma distributed rate categories to account for rate heterogeneity across sites. The gamma shape parameter was estimated directly from the data (1.2). Analysis contemplated a total of 116 nematode and arthropod sequences and reliability for internal branches was assessed using the bootstrapping method (100 bootstrap replicates).

Phylogenetic analysis of the nematode and arthropod TM domain sequences with the human Class 2 B1 members were generated using a similar approach to that described above but only invertebrate receptors for which the 7TM core was completely identified were used (total of 73 nematode and arthropod sequences, (Table S1). NJ analysis contemplated 1000 bootstrap replicates and the rate variation among sites was modeled with a gamma distribution of 4. ML analysis was performed using an estimated proportion of invariable sites (0.019) and 4 gamma distributed rate categories. The gamma shape parameter was fixed (1.4) and analysis performed with 100 bootstrap replicates. Similar analysis was also performed with the deduced amino acid sequence of the putative deuterostome PDF-R-like with the ML method (proportion of invariant sites 0.019, 4 gamma-distributed rate categories, gamma shape 1.019). In all the phylogenetic analysis performed bootstrap values higher than 50% were considered supportive of branching.

### Short-range Gene Linkage

The gene environment of Class 2 B1 family GPCRs was characterized in the nematode *C. elegans* and in the arthropod *T. castaneum*. Gene synteny analysis was performed using the ENSEMBL BioMart comparative tool (http://metazoa.ensembl. org/biomart/martview/) to identify gene sequence homologues within the vicinity of the receptor locus and gene identity was confirmed using BLAST. Genes flanking the representatives of Class 2 B1 receptors in the *T. castaneum* genome were compared with the homologue regions in *D. melanogaster* and *A. gambiae* genomes. Cluster A and B gene members were absent from *D. melanogaster* and *A. gambiae* genomes and to characterize receptor evolution associated with gene loss, the chromosome position of genes closely linked to receptor genes in *T. castaneum* genome were established.

Comparisons of the linkage environment of Class 2 B1 receptors in *C. elegans*, *T. castaneum* and the vertebrates chicken (*Gallus gallus*) and human (*Homo sapiens*) were also performed. Homologues of the genes flanking the *T. castaneum* receptors were procured in chromosomes III and X of *C. elegans* and in the chicken and human chromosomes.

## Results

### Class 2 B1 Members in Nematodes and Arthropods

Sequence homologues of *C. elegans*, *D. melanogaster* and human Class 2 B1 receptors were identified and retrieved from several nematode and arthropod genomes (Table 2). In this study, 6 nematode and 18 arthropod (15 insects, 1 crustacean and 2 arachnid) genomes were analyzed (Table 2, Table S1 and S2) and a total of 121 putative Class 2 B1 receptor genes were retrieved.

**Table 2:** Members of Class 2 B1 GPCRs identified in Nematoda and Arthropoda phyla.

Phyla	Order	Species names	DH44-R	DH31-R	Hec-R	PDF-R	cluster A	cluster B	total
NEMATODA	Rhabditida	*Caenorhabditis elegans*	*n.i.*	*n.i.*	*n.i.*	2	1	*n.i.*	3
	Strongylida	*Haemonchus contortus*	*n.i.*	*n.i.*	*n.i.*	2	1	*n.i.*	3
	Diplogasterida	*Pristionchus pacificus*	*n.i.*	*n.i.*	*n.i.*	1	1	(1)	3
	Tylenchida	*Meloidogyne incognita*	*n.i.*	*n.i.*	*n.i.*	2	*n.i.*	*n.i.*	2
	Spirurida	*Brugia malayi*	*n.i.*	*n.i.*	*n.i.*	2	*n.i.*	*n.i.*	2
	Trichurida	*Trichinella spiralis*	*n.i.*	*n.i.*	*n.i.*	2	1	1	4
									
ARTHROPODA	Diptera	*Drosophila melanogaster*	2	1	1	1	*n.i.*	*n.i.*	5
		*Aedes aegypti*	2	1	1	1	*n.i.*	*n.i.*	5
		*Anopheles gambiae*	2	1	1	1	*n.i.*	*n.i.*	5
		*Anopheles darlingi*	1	1	*n.i.*	1	*n.i.*	*n.i.*	3
		*Culex quinquefasciatus*	2	1	1	1	*n.i.*	*n.i.*	5
	Hymenoptera	*Apis mellifera*	1	1	*n.i.*	1	*n.i.*	2	5
		*Nasonia vitripennis*	1	1	*n.i.*	1	*n.i.*	4	7
		*Atta cephalotes*	1	1	*n.i.*	1	*n.i.*	1	4
	Coleoptera	*Tribolium castaneum*	2	1	1	1	1(1)	2	9
	Lepidoptera	*Bombyx mori*	1	1	1	1	1	*n.i.*	5
		*Danaus plexippus*	1	1	1	1	1	*n.i.*	5
		*Heliconius melpomene*	2	1	*n.i.*	1	1	*n.i.*	5
	Hemiptera	*Acyrthosiphon pisum*	2	2	1	1	*n.i.*	*n.i.*	6
		*Rhodnius prolixus*	1	2	1	1	*n.i.*	1	6
	Phthiraptera	*Pediculus humanus*	1	1	*n.i.*	1	1	1	5
	Cladocera	*Daphnia pulex*	2	1	*n.i.*	1	1	1	6
	Ixodida	*Ixodes scapularis*	4	1	*n.i.*	2	1 (2)	1	11
	Trombidiformes	*Tetranychus urticae*	2	1	*n.i.*	2	1	1	7

**Figure 1:**
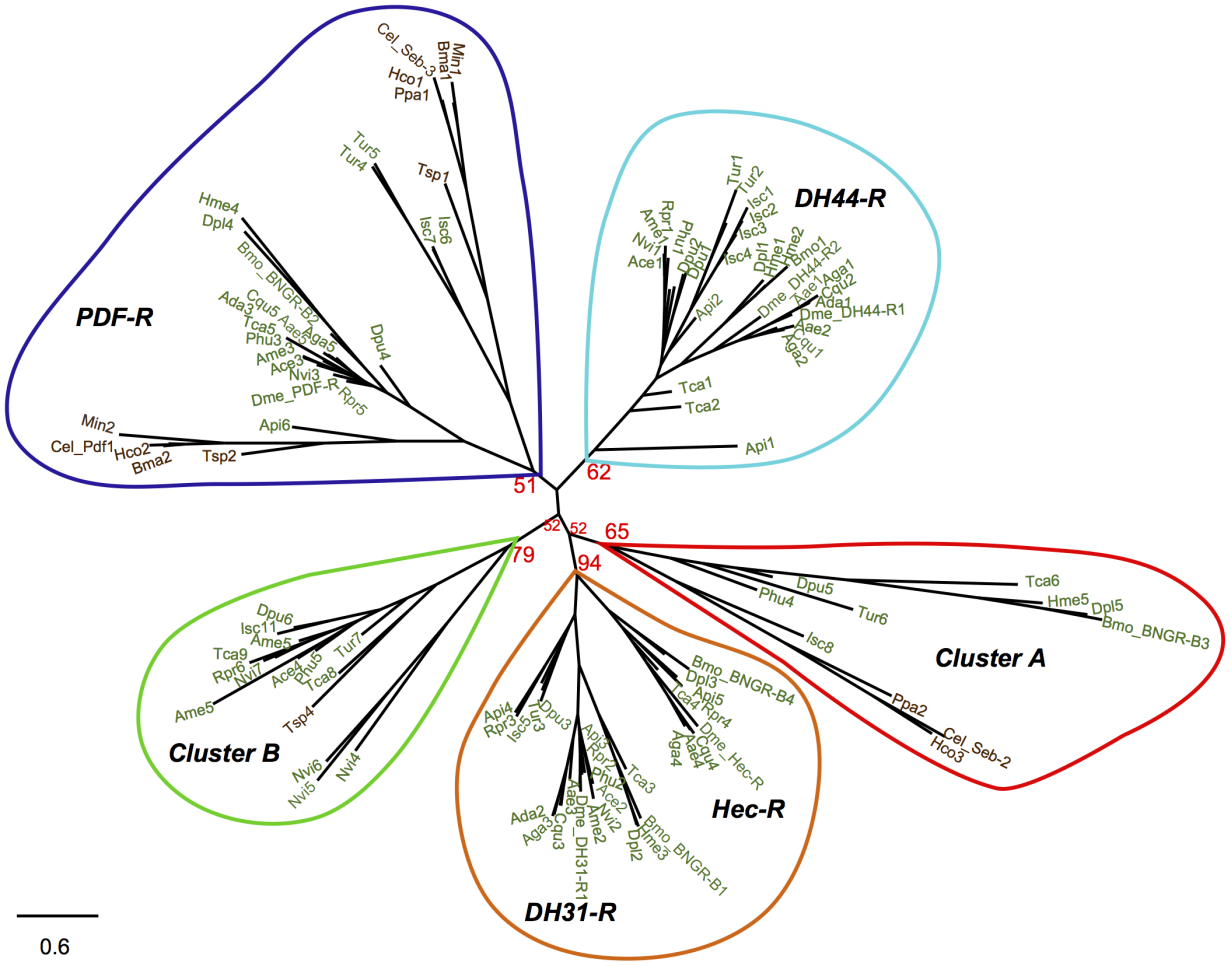
Evolutionary tree of the nematode and arthropod Class 2 B1 receptors. The five distinct groups of the nematode and arthropod Class 2 B1 identified are annotated in color. The phylogenetic tree is constructed with 116 nematode and arthropod receptor sequences using the maximum likelihood method implemented in the PhyML program (v3.0 aLRT) and using the alignment of the conserved TM domains. Four sequences were not included in the analysis due to the incomplete nature of their TMs and they are indicated in Table S1. Reliability of internal branching is assessed using the bootstrapping method (100 bootstrap replicates). For simplicity, only bootstrap support nodes for the main protostome clades are represented. The complete phylogenetic tree is available as Figure S2.

Six receptor groups were identified in this study based on sequence homology with *C. elegans* and *D. melanogaster*. Genes that are not included in phylogenetic analysis (Figure 1) are indicated in brackets, *n.i.*; not identified. Accession numbers available in Table S1.

In nematode genomes 2 to 4 putative Class 2 B1 receptor genes were identified. In the red stomach worm nematode *H. contortus* (Strongylida order) and in the parasitic worm *P. pacificus* (Diplogasterida order) 3 putative Class 2 B1 receptors were identified and sequence similarity searches revealed that they were homologues of the *C. elegans* (order Rhabditida) receptors (Table 2, Table S1). In the plant parasitic nematode *M. incognita* (order Tylenchida) and in the lymphatic filariasis worm *B. malayi* (order Spirurida) 2 putative receptor gene homologues of *C. elegans* Seb-3 and PDF-R were also retrieved and failure to identify the Seb-2 homologue may be a consequence of the incomplete nature of the genome assembly. In the genomes of the mammalian parasite *T. spiralis* (order Trichurida) 4 putative receptor genes were obtained and two PDF-Rs seem to exist (Table 2, Table S1).

In arthropod genomes a variable number of receptor genes were identified ranging from 5 to 9 in insects, 6 in the crustacean, *D. pulex* (order Cladocera) and in the Arachnids *I. scapularis* (order Ixodida) and *T. urticae* (order Trombidiformes) 11 and 7 genes were retrieved, respectively (Table 2, Table S1). In insects, 5 Class 2 B1 receptor genes were present in fruit fly *D. melanogaster* (order Diptera) and also in the mosquito Culicidae representatives (except *A. darlingi*). In other insect genomes a variable number of Class 2 B1 receptors were obtained. In the genomes of the Hymenoptera insect order the honeybee *A. mellifera*, the jewel wasp *Nasonia vitripennis* and the leafcutter ant *A. cephalotes* contained 5, 7 and 4 Class 2 B1 genes, respectively. In the three representatives of the Lepidoptera order the silkworm *B. mori,* monarch butterfly *D. plexipus* and the postman butterfly *H. melpomene* 5 genes were retrieved and, in the human lice *P. humanus* (Phthiraptera order) a similar gene number was also found. In the two Hemiptera genomes analyzed, *A. pisum* and *R. prolixus*, 6 putative genes were found and the red flour beetle *T. castaneum* (order Coleoptera) is the insect with the greatest number of representatives and 9 potential receptor genes were identified (Table 2, Table S1).

### Phylogeny and Sequence Comparisons of the Nematode and Arthropod Class 2 B1 Members

Phylogenetic analysis of Class 2 B1 receptors grouped the nematode and arthropod receptors according to their similarity (Figure 1). At least 5 Class 2 B1 phylogenetic receptor clusters with bootstrap support were identified and 2 represent novel receptor phylogenetic clades. The novel clades were designated Class 2 B1 cluster A and Class 2 B1 cluster B since, at present, no function has been attributed. Cluster A contained both nematode and arthropod receptors and cluster B contained predominantly arthropod receptors. Representatives of cluster A and cluster B were not identified in Diptera indicating that these genes may have been lost from their genomes (Table 2, Figure 1).

### The Novel Nematode and Arthropod Class 2 B1 Members

A total of 15 receptors were identified in receptor cluster A and included the nematode *C. elegans* Seb-2 and the arthropod *B. mori* BNGR-B3 and also the invertebrate receptors of the phylum Nematoda (*H. contortus*, *P. pacificus* and *T. spiralis*) and Arthropoda (*T. castaneum*, *H. melpomene*, *D. plexippus, P. humanus*, *D. pulex, T. urticae* and *I. scapularis*) (Table 2, Figure 1, Table S1). In contrast, members of this family were not identified in the representative genomes of Hymenoptera and Hemiptera orders suggesting that selective gene deletion occurred during the insect radiation. Sequence comparison of the predicted TM domain regions revealed that *C. elegans* Seb-2 shares 42–58% amino acid (aa) sequence similarity with the members of arthropod cluster A and 64% and 78% with the nematode homologues from *H. contortus* (Hco3) and *P. pacificus* (Ppa2) genomes, respectively (Table S3). Within arthropods, members of the cluster A were also conserved and aa sequence similarity varied from 41% between *B. mori* (BNGR-B3) and *P. humanus* (Phu4) to 92% between the postman butterfly *H. melpomene* (Hme5) and *D. plexippus* (Dpl5) (Table S3). Duplicate cluster A receptor members were found in some arthropod genomes but no paralogues were retrieved from the nematode genomes analyzed. In the *T. castaneum* genome two cluster A members (Tca6 and Tca7) were identified however Tca7 gene was very incomplete and only TM1 was characterized, but similarity of the predicted sequences revealed they are highly related. The *I. scapularis* genome contained three putative cluster A representatives, Isc8, Isc9 and Isc10 and Isc8 has the most complete sequence (Figure S1).

Class 2 B1 cluster B receptors included 2 from nematodes and 15 from arthropods. Nematode receptor homologues were found in the *P. pacificus* and *T. spiralis* genomes and the deduced *P. pacificus* receptor gene (PPA19772) was very incomplete and only contained the N-ted region and lacked TM domains but in *T. spiralis* (Tsp4) 5 TM domains were identified that share 35–47% aa sequence similarity with the arthropod homologues (Table S4). In arthropods, cluster B receptors were retrieved from *T. castaneum, A. mellifera, N. vitripennis, A. cephalotes, P. humanus, R. prolixus, D. pulex*, *T. urticae* and *I. scapularis* genomes (Table 2). No cluster B receptor homologues were identified in representatives of the insect Lepidoptera order. In arthropod genomes, duplicate cluster B receptor genes were found in *A. mellifera* (Ame4 and Ame5, which share 64% aa sequence similarity) and in *T. castaneum* (Tca8 and Tca9 that share 70% aa sequence similarity) (Table S4) and four receptors were retrieved from the *N. vitripennis*. Sequence comparisons revealed that within arthropods, aa sequence similarity varied from 57% between *A. mellifera* (Ame5) and *T. castaneum* (Tca8) to 84% between *D. pulex* (Dpu6,) and *P. humanus* (Phu5) (Table S4).

Sequence comparisons of the novel cluster A and B receptors with the other Class 2 B1 receptors in invertebrates revealed that members of cluster A were the most divergent (37–44% similarity) while cluster B genes shared 50 to 65% aa similarity with the invertebrate DH44-R, DH31-R, Hec-R and PDF-R genes (Table S5).

### Homologues of the *C. elegans* and *D. melanogaster* Receptors

Searches in arthropod genomes also identified putative Class 2 B1 receptors of the invertebrate DH44-R, DH31-R, Hec-R and PDF-R subfamilies (Table 2, Figure 1 and Table S1). In contrast, in nematodes, homologues of the *D. melanogaster* DH44-R, DH31R and Hec-R subfamilies were not retrieved suggesting that they are specific to arthropod genomes. Homologues of the *C. elegans* Seb-3 and PDF-R were identified in the genomes of all other nematodes analyzed and clustered with the arthropod PDF-Rs. The PDF-R cluster contained representatives from most taxa (Table 2). The nematode PDF-Rs shared 36–62% aa similarity with the arthropod homologues and, within the arthropods these receptors shared 62–71% aa similarity (Table 3). Within the invertebrate PDF-R cluster, the *C. elegans* Seb-3 and other nematode sequence homologues and the duplicate arachnidan genes, grouped on an independent branch suggesting they may be part of a novel receptor cluster (Figure 1).

**Table 3:** Percentages of amino acid sequence similarity of *T. castaneum* receptors with the nematode and arthropod homologues.

	DH44-R	DH31-R	Hec-R	PDF-R	Cluster A	Cluster B
**NEMATODE**	–	–	–	36–62%	37–40%	41–44%
**ARTHROPOD**	67–83%	53–78%	79–84%	62–71%	54–64%	57–78%

*T. castaneum* receptors were chosen for sequence comparisons since a representative of each Class 2 B1 subfamily was identified in its genome. For sequence similarity calculations only the nematode and arthropod receptors with more than 6 TM domains identified were considered (see Figure S1).

The arthropod homologues of *D. melanogaster* DH31-R and Hec-R were always clustered in phylogenetic analysis and shared the highest sequence similarity suggesting that they have evolved from an ancestral receptor that was already present in an early arthropod lineage (Figure 1 and Table S5). In the genomes of the arthropods, *A. darlingi*, *A. mellifera*, *N. vitripennis*, *A. cephalotes*, *H. melpomene*, *P. humanus*, *D. pulex*, *T. urticae* and *I. scapularis* no putative Hec-R genes were identified and a single DH31-R gene was retrieved with the exception of the representatives of the Hemiptera order (*A. pisum* and *R. prolixus*) that possessed two DH31-R genes. The arthropod DH31-R members were 53–78% similar and the Hec-R genes were the most conserved Class 2 B1 receptors and their members’ shared 79–84% sequence similarity (Table 3). The arthropod homologues of the *D. melanogaster* DH44-R (R1 and R2) paralogues also clustered and were 67%–83% similar and gene duplicates were also identified in the majority of the arthropod genomes analyzed and in the *I. scapularis* genome 4 genes may exist (Table 2, Figure 1). In contrast, in *A. darlingi*, *A. mellifera*, *N. vitripennis, A. cephalotes*, *B. mori*, *D. plexippus, R. prolixus* and *P. humanus* only a single receptor gene was identified and the failure to identify a duplicate receptor may be due to their incomplete genome assemblies.

### Homology for the Human Receptors

**Figure 2:**
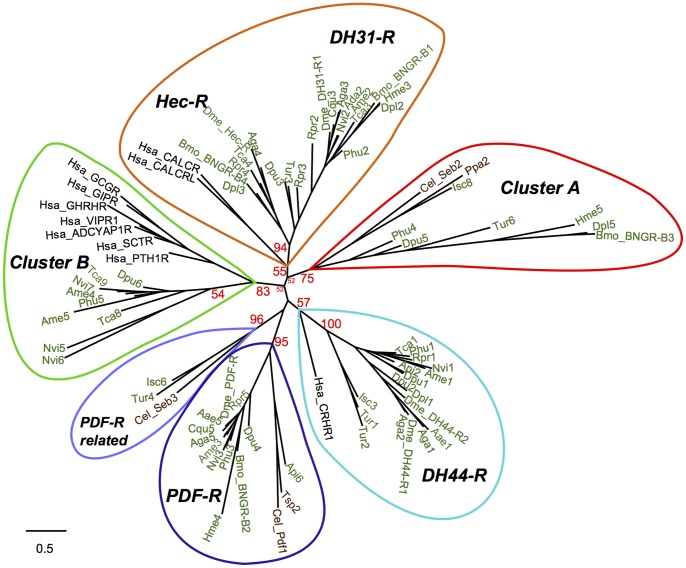
Evolutionary relationships of the nematode and arthropod Class 2 B1 members with the human homologues. The metazoan receptor groups identified are annotated in color. The phylogenetic tree is constructed using the maximum likelihood method implemented in the PhyML program (v3.0 aLRT). Reliability of internal branching is assessed using the bootstrapping method (100 bootstrap replicates). Analysis is based on the amino acid sequence alignment of the TM regions of Class 2 B1 receptors and is performed using only receptors with the full complement of seven TM domains in human, nematodes and arthropods (total of 73). Sequences omitted from the analysis are indicated in Table S1. For simplicity, only bootstrap support for the main receptor nodes is shown. The complete phylogenetic tree is available as Figure S3.

The nematode and arthropod receptors including the novel members (cluster A and B) were compared with the human Class 2 B1 receptors to assign potential sequence homologies (Figure 2). The nematode and arthropod cluster B receptors group with the human GPS-receptor group with bootstrap support suggesting that they are highly related and share common ancestry. The human CALCR and CALCRL receptor members grouped with the arthropod DH31-R and Hec-R with which they share the highest similarity (66–72%, Table S6) and the human CRHR receptors with the arthropod DH44R. In addition, inclusion of human CRHR in phylogenetic analysis clearly divided the PDF-R cluster into two receptor phylogenetic clades and separated the nematode *C. elegans* Seb-3 receptor containing cluster (named in this study PDF-R related cluster) from a second cluster of invertebrate PDF-Rs that contained *C. elegans* and *D. melanogaster* receptors (Figure 2). The nematode and arthropod cluster A were the most divergent receptors and members did not group with any of the human Class 2 B1 receptor subfamilies (Table S6).

### Conserved Gene Environment within *C. elegans* and *T. castaneum*

In *C. elegans*, three Class 2 B1 receptors have been identified and Seb-3 maps to chromosome X while Seb-2 and PDF-R are in close proximity on chromosome III and probably arose from a gene duplication event. Comparisons of nematode chromosomes III and X revealed that Seb-3, Seb-2 and PDF-R share a similar gene environment and share loci such as: collagen genes (let-2, emb-9, col-41 and 90), mitochondrial protein genes (gas-1 and nduf-2.2), sodium-coupled dicarboxylate transporters (nac-1 and 3), beta-N-acetylhexosaminidase genes (hex-3 and 5) and AMP kinase subunit genes (aakb-1 and 2) (Figure S4).

In the beetle genome, cluster A genes (Tca6 and Tca7) map to chromosome LG2 and gene position suggests they may be the result of a tandem gene duplication. Cluster B (Tca8) is found in chromosome LG4 and its duplicate (Tca9) has not yet been mapped. The duplicate DH44-Rs (Tca1 and Tca2) are present in LG4 and LG9, respectively and PDF-R and Hec-R (Tca5 and Tca4) map to LG5. The locus for DH31-R (Tca3) in *T. castaneum* has not yet been determined (Figure S4). The gene environment of some Class 2 B1 receptors in *C. elegans* and in *T. castaneum* contains representatives of other gene families. For example, comparison of the gene environment of the duplicate beetle DH44-R genes reveals that each contains a gene of the nicotinic acetylcholine receptor family (NACHR and NACHR-like) and a representative of the cluster of differentiation 36 (CD36) family (Santa-maria and Snmp2). In the vicinity of the remaining Class 2 B1 loci, representatives of other gene families were also found: cytochrome P450 (Cyp4, CYP6BQ1 and CYP9AD1) on LG2, LG4 and LG5; Forkhead (FKH) family (fd3F, FKH, FKH-like and Sloppy paired 1, SLP-1) in LG2, LG4 and LG5; Homeobox genes (EY, AL, REPO, GSC and TOY) distributed on LG4, LG5 and LG9; Odorant binding protein (OBP) members (OBP-C18 and OBP-C16) on LG2 and LG4 and tyrosine kinase genes (InR, Wsck and RET) on LG4, LG5 and LG9 (Figure S4). The conservation of the gene-linkage environment of Class 2 B1 members in the *C. elegans* and beetle genomes suggests that receptor gene evolution may have involved chromosome segment duplications. It remains to be established if flanking genes of the invertebrate Class 2 B1 receptors are also part of multigene families with gene copies in other nematode and arthropod chromosomes.

### Comparisons across Arthropods

**Figure 3:**
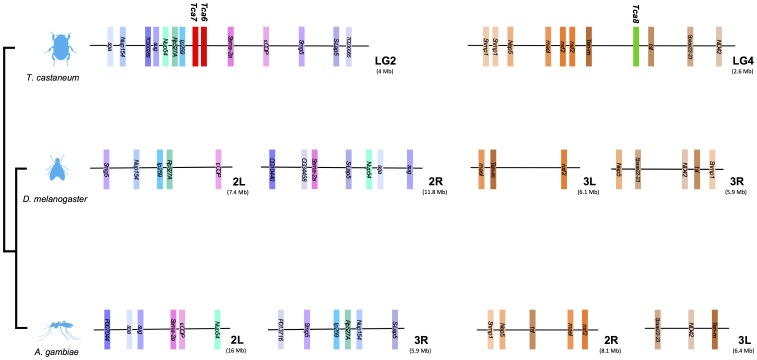
*T. castaneum* cluster A and B short-range gene environment in *D. melanogaster* and *A. gambiae* chromosomes. The immediate gene environment of *T. castaneum* cluster A (Tca6 and Tca7) members in chromosome 2 and cluster B (Tca8) members in chromosome 4 was compared with the homologue genome regions in *D. melanogaster* and *A. gambiae.* Genes are represented as colored blocks to facilitate visualization and receptor loci are annotated in bold and are colored in accordance with receptor clustering (Figure 1). *T. castaneum* gene names, when unknown, are given based upon *D. melanogaster* annotation (www. Flybase.org). Solid horizontal lines represent chromosome fragments and distances compared (Mb) are indicated. The relative position of the gene in the chromosomes is shown. Only genes common in all species are represented.

**Figure 4:**
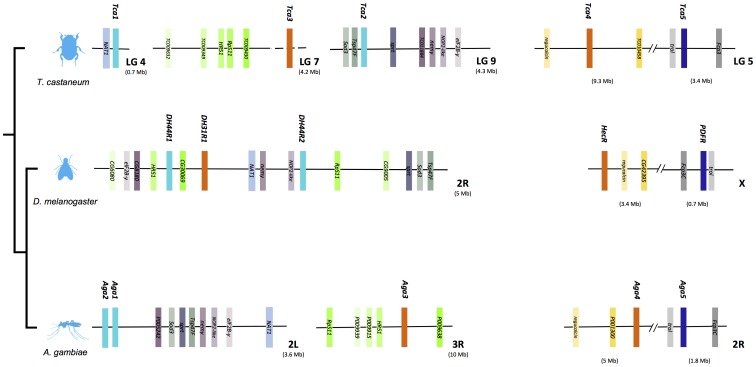
Short-range gene linkage of the arthropod Class 2 B1 receptor genes. Genes are represented by colored blocks to facilitate identification. Class 2 B1 receptor genes are annotated in bold and gene blocks colored according to the tree clustering of Figure 1. Gene names, when unknown, are given based upon the *D. melanogaster* annotation (www. Flybase.org). Solid horizontal lines represent the chromosome fragment and double bars within the chromosome lines represent interruption and the gene distances compared are indicated (Mb). For simplicity only genes that are common in all the species are represented and their relative positions in the chromosomes are indicated.

Comparison of Class 2 B1 receptors gene environment between the insects *T. castaneum, D. melanogaster* and *A. gambiae* revealed conserved gene order and synteny. Considerable chromosome rearrangements were observed for the receptor homologue genome regions between organisms and this suggests that Class 2 B1 receptor genes were under different evolutionary pressures (Figure 3 and 4).

### Potential Loss of Cluster A and B Loci in Diptera

Sequence homologues of the novel cluster A and cluster B receptors are absent from *D. melanogaster* and *A. gambiae* genomes although genes in linkage with the receptors in other species were identified (Figure 3). A similar complement of genes to those surrounding the *T. castaneum* cluster A in LG2 was also found in *D. melanogaster* and *A. gambiae* but were clustered on two different chromosomes. The *T. castaneum* genes for *Ip259* (Intronic Protein 259) and *Sema-2a* (Semaphorin-2a) that are linked to beetle cluster A receptor members are localized in *D. melanogaster* 2R and 2L and in *A. gambiae* 3R and 2L, respectively a trend that was also observed for other genes for the genome regions analyzed (Figure 3). Similarly, the genes flanking cluster B genes in *T. castaneum* chromosome 4 were divided between two chromosomes in *D. melanogaster* (3R and 3L) and *A. gambiae* (2R and 3L) (Figure 3). For example, homologues of beetle cluster B linked-genes *Ten-m* (Tenascin major) and *tsl* (torso-like) map to *D. melanogaster* 3R and 3L and to *A. gambiae* 3L and 2R, respectively and other genes within the beetle LG4 region follow the same pattern (Figure 3). This suggests that the ancestral insect genome region that originated LG2 and LG4 that contain cluster A and cluster B in *T. castaneum* underwent considerable rearrangements and this resulted in the formation of at least two different chromosomes in Diptera (Figure 3).

### Gene Linkage Conservation with the *D. melanogaster* and *A. gambiae* Receptor Homologues

Conservation of gene environment was observed between the *T. castaneum* and the *D. melanogaster* and *A. gambiae* receptor homologue regions (Figure 4). Comparisons of the DH44-R gene environment revealed that between the 3 insects at least 8 genes were shared: *nemy* (no extended memory); *NOP-2-like* (nucleolar protein homolog); *sprt* (Sepiapterin reductase); *Sod3* (Superoxide dismutase 3); *tsp47F* (Tetraspanin 47F); *eIF2B-y* (eukaryotic initiation factor 2B), CG8180 and *NAT1*. In *T. castaneum* the duplicate DH44-Rs (Tca1 and Tca2) genes and are localized in LG4 and LG9 while in *D. melanogaster* both genes share the same chromosome as DH31-R (chromosome 2R) and in *A. gambiae* both DH44-Rs map in close proximity on chromosome 2L and their genome position in the latter species suggests that they are the result of a tandem gene duplication (Figure 4).

The Hec-R and PDF-R genes share the same chromosome in *T. castaneum*, *D. melanogaster* and *A. gambiae* genomes (Figure 4). In *T. castaneum* both genes map to LG5, in *D. melanogaster* they are located on chromosome X and in the *A. gambiae* on chromosome 2R. Homologues of the *D. melanogaster* regucalcin and CG4239 were found in close proximity to arthropod Hec-R loci and *Fcp3C* (Follicle cell protein 3C) and *trol* (terribly reduced optic lobes) genes are close to PDF-R (Figure 4). This suggests, that in contrast to other genome regions harboring Class 2 B1 receptors the structure of these chromosomes evolved under conservative pressures.

Gene environment comparisons in insects also permitted the putative positioning of the *T. castaneum* DH31-R gene (not yet mapped) on LG7 as it shares conserved gene linkage with the receptor genome region in *D. melanogaster* (2R) and *A. gambiae* (3R) (Figure 4). The receptor linked genes were the homologues of the *D. melanogaster* CG6080, *HR51* (Hormone receptor 51), CG30069, *RpS11* (Ribosomal protein S11) and CG9005.

### Conservation of *T. castaneum* Class 2 B1 Receptor Gene Environment in Nematode and Vertebrate Genomes

**Figure 5:**
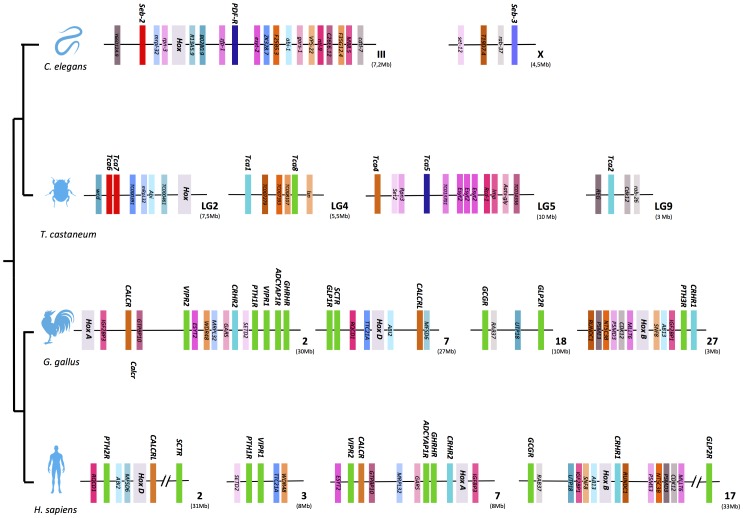
Maintenance of a conserved Class 2 B1 receptor gene environment across metazoans. Metazoan Class 2 B1 receptors are annotated in bold and are colored according to the phylogenetic clustering obtained from Figure 2. Only genes common to all species are represented. Double bars within the chromosome lines represent interruption in the sequence and the length of the genomic region analyzed (Mb) is indicated within brackets. Colored blocks represent genes and when available gene symbols are given. The relative position of the gene on the chromosome is shown. For detailed information please see Table S7.

The beetle Class 2 B1 receptor gene environment was compared to the homologue genome regions in *C. elegans* and at least 20 genes in linkage were identified within the regions analyzed (Figure 5). Sequence homologues of the *C. elegans* and *T. castaneum* genes flanking Class 2 B1 receptor genes (including cluster A and cluster B members) were also identified in the chicken and human chromosomes containing receptor family members (Figure 5).

The gene environment of Class 2 B1 receptor genes in LG2, LG4, LG5 and LG9 in *T. castaneum* was similar to the regions of *C. elegans* chromosome III that encode Seb-2 and PDF-R and chromosome X that contains Seb-3. A similar gene repertoire to those flanking Class 2 B1 receptors in *T. castaneum* and *C. elegans* was found in chicken in the vicinity of the vertebrate members on chromosomes 2 (CALCR, VIPR2, CRHR2, PTH1R, VIPR1, ADCYAP1R, GHRHR), chromosome 7 (GLP1R, SCTR and CALCRL), chromosome 18 (GCGR and GLP2R) and chromosome 27 (PTH3R and CRHR1). A similar situation occurred in humans on chromosome 2 (PTH2R, CALCRL, SCTR), chromosome 3 (PTH1R and VIPR1), chromosome 7 (VIPR2, CALCR, ADCYAP1R, GHRHR1 and CRHR2) and chromosome 17 (GCGR, CRHR1 and GLP2R) (Figure 5, Table S7). For example, sequence homologues of *wcd* (wicked gene) and TC000391 that flank beetle cluster A members (Tca 6 and Tca 7) on LG2 were found on *C. elegans* chromosome III near the PDF-R gene and also on chicken chromosomes 18 and 7 and human chromosomes 17 and 3 in the proximity of Class 2 B1 receptor genes. Sequence homologues of the beetle cluster B gene environment on LG4 were found in the nematode chromosome III and in the chicken 2 and 27 and human 3 and 17 chromosomes (Figure 5) and gene homologues of the closely linked beetle TC008107 gene are localized near the GPS-receptor gene members in vertebrate chromosomes.

In the nematode and beetle LG/chromosomes, members of the invertebrate Hox gene family clusters in close proximity with Class 2 B1 receptor genes. In *C. elegans* the Hox cluster is localized on chromosome III and in the beetle on LG2 and in vertebrate genomes three of the four Hox cluster (HoxA, HoxB and HoxD) loci are on chicken chromosomes 2, 27 and 7 and human chromosomes 7, 17 and 2 and map in proximity to Class 2 B1 receptor genes. This suggests that the metazoan Class 2 B1 and Hox gene clusters have undergone similar evolutionary events and that the association of the Class 2 B1 and Hox gene cluster is ancient and existed prior to the protostome-deuterostome divergence (Figure 5).

### Conservation of Receptor N-ted Regions in Metazoans

**Figure 6:**
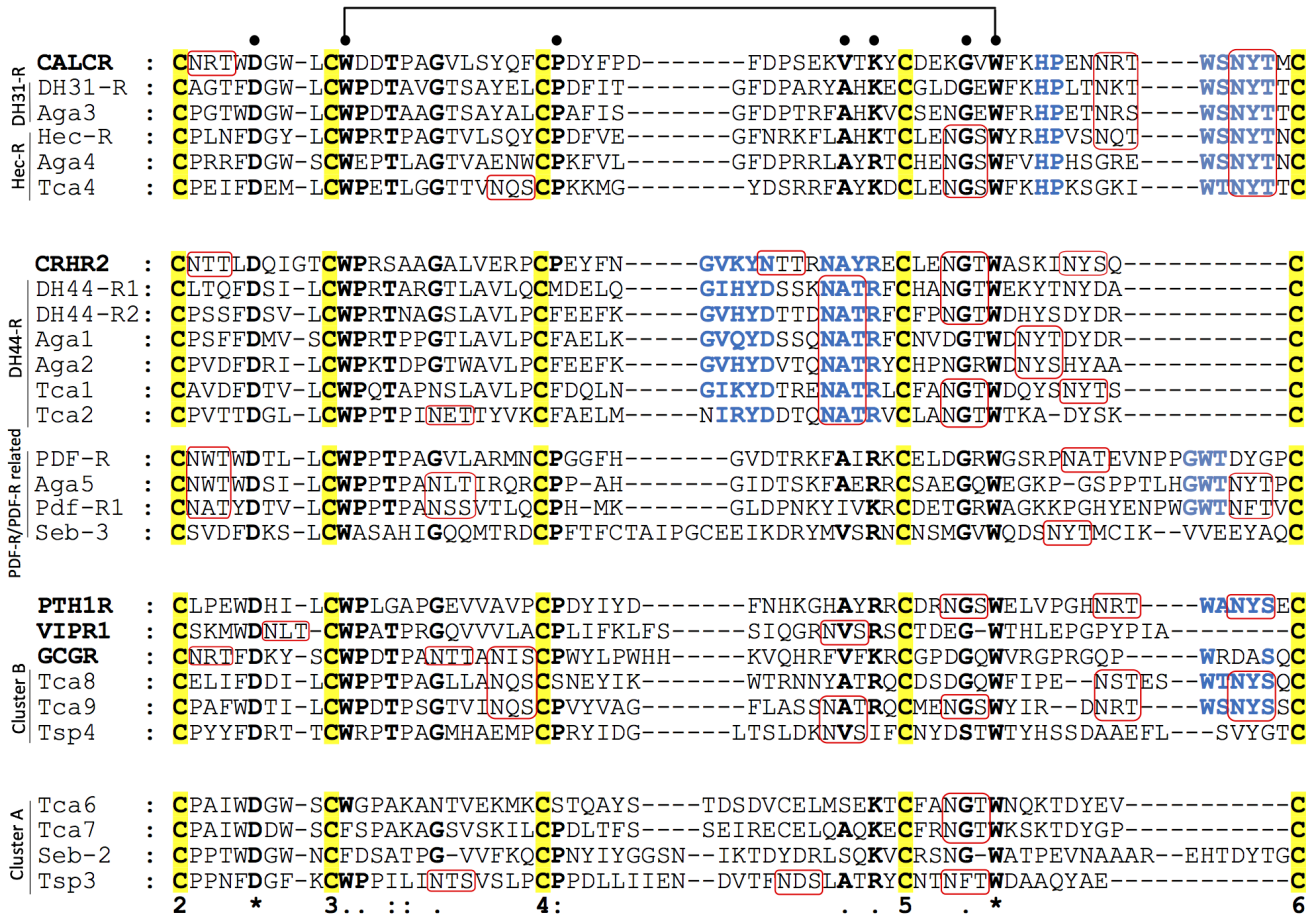
Comparison of the nematode, arthropod and human Class 2 B1 N-ted domains. The protostome receptors are compared with the human receptor homologues identified by phylogenetic analysis. The N-ted receptor region chosen for comparison is flanked by the conserved cysteines, C2 and C6 identified in human receptors. Cysteines are numbered in decreasing order according to their conserved position in relation to the predicted TM1. Conserved amino acid residues across the different receptor families are annotated in bold and conservation within each receptor family is colored in bold and blue. Complete residue conservation is annotated with a “*”, partial conservation with “.” and the position of the amino acids present in most of the receptor families with “:”. Predicted N-glycosylation sites are boxed in red. Accession numbers of the sequences used are in Table S2.

The N-ted regions of the nematode and arthropod receptors were compared with the human homologues and conserved motifs suggestive of potential similarities in ligand-receptor interactions were identified (Figure 6). Five conserved cysteine (C) residues and putative N-glycosylation consensus sites essential for vertebrate Class 2 B1 receptor function [19], [38]–[40] were also identified in the nematode and arthropod receptor N-ted domains suggesting that the conformation of the receptor extracellular domain has been conserved and maintained during evolution.

Key amino acids involved in intramolecular interactions of mammalian receptor N-ted, such as the aromatic indole ring formed by the Tryptophan (W) residues within the C-W-P (P - proline) and G-x-W motifs (G-glycine; x- any amino acid), the basic residues Arginine (R)/Lysine (K), and the hydrophobic residues Valine (V)/Alanine (A) that are localized within the W aromatic ring [19], [39], [40] and between C4 and C5 are also conserved in the majority of invertebrate Class 2 B1 receptors (Figure 6). In addition, aspartic acid (D) located after C2 and involved in structural stabilization of the vertebrate receptors [41], [42] is also present in the nematode and arthropod homologues. The P adjacent to C4, important in vertebrate N-ted hydrophobic interactions, is conserved in all invertebrate receptor subfamilies with the exception of the arthropod DH44-R group that has undergone a specific amino acid mutation (Figure 6).

Comparisons of the human, nematode and arthropod receptors revealed that the DH31R/Hec-R members’ share with the human CALCR a conserved amino acid motif W-S/T-N-Y-T (W-Serine/Threonine-Asparagine-Tyrosine-T, respectively) and a conserved N-glycosylation site (N-x-T) located between C5 and C6, with the exception of Aga4 and Tca4 regions. In addition, the motif Histidine (H)-P between the conserved C5 and C6 positions was also conserved (Figure 6). The *T. castaneum* cluster B receptors also shared a similar motif, W-x-N-Y-S (x-any amino acid), with the human PTH1R but it is absent from the nematode *T. spiralis*.

Within the arthropod DH44-R family and human CRHR2 conserved sequence motifs were also identified and included G-I/V-x-Y-D/N (I – Isoleucine; x-any amino acid) and the N-A-T-R (which in arthropods contains a potential N-glycosylation site) localized between the C4 and C5 that are absent from other receptor subfamilies. The protostome PDF-Rs contain a motif G-W-T near C6 that is absent from the potential human homologue and from the *C. elegans* PDF-R related member (Seb-3). In contrast, no specific N-ted conserved motifs were identified in the nematode and arthropod cluster A representatives that were compared.

Overall, common structural and functional motifs characteristic of human Class 2 B1 receptors were identified in the N-ted domain of invertebrate receptors suggesting that they were under strong conservative pressure before and subsequent to the protostome-deuterostome divergence.

## Discussion

**Figure 7:**
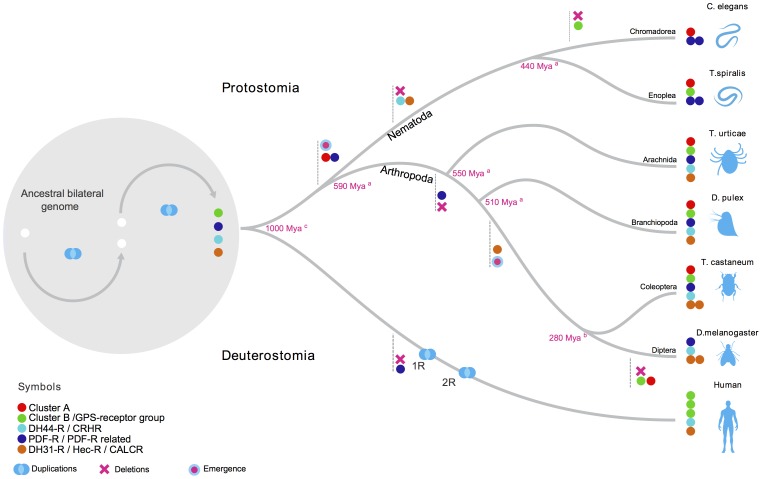
Proposed evolutionary model of the metazoan Class 2 B1 receptor genes. The five major metazoan Class 2 B1 receptor gene phylogenetic clusters are represented by filled colored dots according to their proposed common origin in the bilateral genome (please refer to symbols list). For simplicity, the species-specific gene duplications/deletions of the members within each receptor family are not represented. We propose that the cluster A ancestral gene emerged early in the nematode and arthropod radiation while cluster B is proposed to already be present in the bilateral genome. The ancestral PDF-R/PDF-R-related cluster gene duplication occurred prior to the nematode-arthropod divergence and in the arthropod lineage selective gene deletions occurred. The PDF-R gene has probably been deleted from arachnidan while PDF-R-related gene has been eliminated from the other arthropod genomes. The ancestral DH31-R/Hec-R gene has been deleted in the nematode lineage and Hec-R gene emerged in the insect lineage. Major divergence time points proposed during metazoan evolution in millions of years ago (Mya) are indicated and are taken from; a [64], b [65] and c [66]. The two rounds of genome duplication (1R and 2R) in the deuterostome radiation are represented. The PDF-R-related gene has probably been deleted from the chordate lineage prior to 1R. The figure is not designed to scale.

The vertebrate Class 2 B1 GPCRs are characterized by their unique structural motifs and functional properties such as: *i)* the existence of large N-ted domains, *ii)* the presence of highly conserved cysteine residues and N-glycosylation sites in N-ted; and *iii)* activation of the receptors only by peptide hormones [2], [8], [17]. The N-ted domain of Class 2 B1 receptors interact with peptide ligands and contain amino acid motifs characteristic of each of the five subfamilies [10], [17]. In invertebrates, Class 2 B1 GPCRs have also been identified but their description is mainly limited to *C. elegans* and *D. melanogaster*, which are not good representatives for studies of gene diversity in the nematode and arthropod phyla as species-specific gene rearrangements occurred especially in the members of the dipteran order which have suffered the highest gene molecular evolutionary rate and orthologue gene losses in insects [43]–[45]. In the present study data from genomes of several phylogenetically distinct nematodes and arthropods is used to reevaluate the evolution of Class 2 B1 GPCRs and resulted in the identification of several novel receptor subfamilies. The receptor members of cluster A are specific to nematodes and arthropods and have not previously been identified. Cluster B receptors previously designated as PTHR-like [30] are reclassified as homologues of the vertebrate GPS-receptor group [31] and are identified in nematodes for the first time as up until now they have only been described in arthropods and deuterostomes. Two receptor clusters for PDF are characterized in nematodes and arthropods and designated PDF-R and PDF-R related. No representatives of the novel receptor clusters are present in Diptera. Although relatively few nematode and arthropod genomes are analyzed in relation to the vast diversity of species that exist, the results reveal that the novel Class 2 B1 receptors share a common origin with the previously described metazoan members (Figure 7).

### Evolution of Nematode and Arthropod Class 2 B1 GPCRs

The nematode and arthropod Class 2 B1 receptor gene family members have undergone a number of different evolutionary trajectories as revealed by the variable number of genes identified in distinct taxa. Overall six receptor clusters, which include the orthologues of the *D. melanogaster* DH44-R, DH31-R/Hec-R, protostome PDF-R and three novel receptor clusters (cluster A, cluster B and PDF-R-related cluster) are identified and gene number and sequence analysis reveals they underwent distinct evolutionary trajectories in the nematode and arthropod radiations. In general, nematode genomes contain fewer genes than arthropods and their lower gene content is suggested to result from large and spontaneous gene deletions, which have been associated with the nematode life style and appears to have affected the evolution of GPCR genes [46], [47]. Homologues of the arthropod DH44-R, DH31-R and Hec-R are not found in nematode genomes and they were potentially eliminated from the nematode radiation. A recent study of another GPCR family, the rhodopsin family, revealed that parasitic nematode genomes contain fewer rhodopsin GPCR genes when compared to free-living nematodes in which specific gene expansion has occurred [32]. That parasitic nematode genomes possess lower gene content than other nematodes has also been demonstrated for other gene families [48], [49]. However this does not appear to be the case for the nematode Class 2 B1 GPCR genes as gene number in the parasitic and nonparasitic genomes analyzed is similar and the functional significance of this observation remains to be established.

In arthropods, rhodopsin GPCR evolution was recently described and GPCR gene evolution was proposed to be affected by species-specific events [32]. In the present study, the number of Class 2 B1 receptor genes in the different arthropod genomes analyzed is also variable but similar gene numbers are identified in representatives of the same order (Table 2). The non-identification of cluster A and B genes in *D. melanogaster* and also in Culicidae and the loss of the associated linkage groups suggest that receptor gene deletion may have affected all dipterans as a consequence of specific chromosome rearrangements. In Diptera, orthologous gene loss has previously been described and comparative evolutionary studies of insects and vertebrates revealed that Diptera genomes have suffered the highest gene loss and placental mammals the least gene loss during evolution [45]. Higher gene loss in members of the Diptera order seems to be a consequence of their accelerated molecular evolutionary rate as their genomes are proposed to have evolved two to three times faster than other insects and vertebrates [45]. Overall, the results of the present study support the notion that *T. castaneum* contains a gene repertoire more similar to the bilateral ancestral genome and is probably a better genome model for studies of arthropod gene evolution [43].

Class 2 B1 receptor evolution in arthropods has involved not only gene loss but also gene duplication early and prior to their expansion. The DH31-R and Hec-R potentially emerged from an early gene duplication event and duplication of DH44-R in arthropods to generate DH44-R1 and DH44-R2 genes is potentially a species-specific event (Figure 1). The presence of species-specific gene duplications in arthropods leading to multi-copy orthologous groups has been linked to a high incidence of chromosome fusion and gene rearrangements [43]–[45], [50]. An example of this is the evolution of the arthropod DH44-R genes. With the exception of *I. scapularis*, in the majority of the genomes of the species analyzed, two DH44-R genes are identified and in *D. melanogaster* and *A. gambiae* the duplicate receptor genes map to the same chromosome and the close proximity of the duplicates in *A. gambiae* suggest they are the result of a tandem gene duplication event. In contrast, in the beetle, *T. castaneum*, the DH44-R duplicates are localized on two distinct chromosomes and cluster independently of the *D. melanogaster* and *A. gambiae* genes in phylogenetic analysis suggesting that, the selective pressures within the different insect groups were distinct (Figure 1 and Figure 4).

Curiously in the beetle genome, Class 2 B1 receptors map in close proximity with multigene family members, for example, the P450 family, odorant-binding proteins and also the Hox gene family. In *T. castaneum*, expansions of the cytochrome P450 family and odorant-binding protein family genes are suggested to be driven by selective pressures as they adapted to their habitat [43], [51]. The localization of vertebrate Class 2 B1 receptor genes on chromosome fragments bearing members of the Hox gene family was recently shown and was suggested to already exist in the gnathostome ancestral chromosome [52]. The present study reveals that Class 2 B1 receptor genes and the Hox gene cluster were already in linkage in the arthropod and nematode chromosomes and indicates it existed prior to the protostome-deuterostome divergence and was presumably present in the ancestral bilateral genome.

Our findings support the results of a recent broad molecular study that characterized the evolution of GPCR peptidergic signaling in bilaterians [31]. In the former study the authors performed a comprehensive phylogenetic analysis on the evolution of 13 peptide hormone binding GPCRs families and by comparing data from representative genomes of major metazoan phylum they identified 5 main Class 2 B1 receptor groups, four are homologues of DH44-R, DH31-R/Hec-R, PDF-R and cluster B and one is uncharacterized (unchar-4) receptor group [31]. In the present more detailed study of Class 2 B1 GPCR evolution in ecdysozoa the same 4 classes were identified but an additional receptor cluster, named here cluster A, was also found.

The novel receptor A cluster is characteristic of nematode and arthropod genomes and includes the *C. elegans* Seb-2 gene, previously suggested to be a potential homologue of the arthropod DH31-R [31]. In our study, Seb-2 always clustered independently of the arthropod/deuterostome DH31R/CALCR clusters and the bootstrap support in phylogenetic analysis indicates that the nematode Seb-2 and the other nematode and arthropod sequence homologues are members of the novel Class 2 B1 receptor cluster. Further support for this idea comes from comparisons of the Seb-2 receptor N-ted domain (Figure 6) that reveals Seb-2 lacks the conserved arthropod/deuterostome DH31R/CALCR motifs. Seb-2 linkage analysis indicates conservation with genome regions containing Tca6 and Tca7 (cluster A receptors). The higher number of nematodes and arthropods used in the current study also permitted clear positioning of the nematode receptors (Cel-Seb-3 and Ppa1) previously consigned to unchar-4 group [31] in the newly described PDFR-related cluster that has evolved from the same ecdysozoa ancestral receptor as PDFR and includes other nematode and arthropod representatives (Figure 1 and 2).

Although only nematode and arthropod genomes were examined, the present study indicates that invertebrate Class 2 B1 receptors share a conserved evolutionary origin with the vertebrate homologues. The arthropod DH31-R/Hec-R are evolutionary closely related with the human CALCR and DH44-R with the human CRHR (Figure 7). Recently, the *T. castaneum* and *A. mellifera* Class 2 B1 GPCRs that grouped in cluster B were defined to be the homologues of the vertebrate PTHRs [30]. Our results as well those obtained by Mirabeau and Joly [31] only partially support this classification and provide evidence that they also share early emergence with the GCG and SCT receptor group (Figure 2, Figure 7). No gene representatives of cluster A appear to exist in vertebrates.

To establish if a cluster A gene member is present in other deuterostomes (excluding human) a preliminary search was performed. No putative cluster A receptor gene was identified in the early chordate genomes of Ciona (*C. intestinalis*), amphioxus (*B. floridae*), sea urchin (*S. purpuratus*) or any vertebrate suggesting that cluster A members are exclusive to protostomes and may have emerged after their divergence from deuterostomes (Figure 7). PDF-R and PDF-R related cluster members are also proposed to be exclusive to protostome genomes and have many functions assigned. In crustaceans and insects PDF-R is involved in pigment change and regulation of biological rhythms and the nematode PDF-R related is involved in locomotion [53]. In early deuterostome genomes (echinoderm, hemichordate and cephalochordate) putative homologues of the nematode and arthropod PDF-R/PDF-R-related receptors were identified (this study and Mirabeau and Joly [31]) and they are apparently absent from vertebrate genomes (Figure S1 and Figure S5). This indicates that a putative ancestral PDF-R/PDF-R-related receptor gene was present prior to the protostome-deuterostome divergence but was subsequently lost during evolution (Figure 7). The deduced amino acid sequence of the sea urchin (*S. purpuratus*) PDF-R is 60% similar to the *C. elegans* and *T. castaneum* (Table S6) homologues and its function as for the other early deuterostome homologues remains to be established, as does that of the ligand.

### Conservation of Class 2 B1 GPCR Function in Metazoan

In vertebrates, Class 2 B1 members are characterized by the presence of large N-terminal domains, which are the main receptor structures involved in ligand binding. This contrasts with other GPCRs in which other parts of the receptor structure including the TM and extracellular loops are also involved in ligand binding. In fact, a recent two-domain model for Class 2 B1 ligand-receptor binding interactions includes the receptor TM domains [10], [17]. According to the two-domain model, the central and C-terminal parts of the peptide ligand are trapped by the N-ted of the receptor leaving the peptide N-ted to interact with the receptor TM domain. In protostomes, despite the incomplete nature of the majority of the N-ted regions of the receptors identified it was possible to establish that generally the region is similar in length to the vertebrate homologues and that the conserved amino acid residues/motifs are those involved in ligand-binding in vertebrates. This suggests that despite the low amino acid sequence conservation between ligands from invertebrates and vertebrates the model for receptor activation is similar.

In *D. melanogaster* with the exception of Hec-R, which has a role in male courtship behavior [29], but still has no ligand specific peptide receptor agonists have been identified. The function of Class 2 B1 family members have been described mainly in arthropods and have been proposed on the basis of sequence and function to be related to the vertebrate ligand-receptor endocrine pairs [22], [27], [32], [54]–[56] (Table 1). Diuretic hormone 44 (DH44) in arthropods is the putative homologue of mammalian Corticotropin Releasing Hormone (CRH) and activates both DH44-R1 and DH44-R2 and the signaling pathway is suggested to be similar to the vertebrate CRH signaling systems [21]. These peptides are involved in osmotic balance and diuresis in insects through binding to their receptors in the Malpighian tubules [21], [22], [57]–[59]. The insect neuropeptide DH31 is suggested to be a structure homologue of vertebrate CALCA peptides, and in common with DH44 affects water transport in the Malpighian tubules of several insects [60]. DH31 has low similarity with the vertebrate CALCA and the only conserved motif in the C-terminal region, Gly-X-Pro, is crucial for both invertebrate and vertebrate peptide activity [55], [60], [61]. The arthropod PDF peptide and its homologue in the nematode *C. elegans*, are the principal neurotransmitters regulating circadian locomotor rhythms in protostomes [26], [28], [53]. In *D. melanogaster*, PDF binds to its specific PDF-R and dNF1 (drosophila Neurofibromatosis) peptide potentiates PDF signaling suggesting that in protostomes, Class 2 B1 receptor modulation can occur by membrane auxiliary proteins as occurs in vertebrate [28], [62]. In mammals, PAC_1_ and VPAC receptors are involved in the regulation of the circadian system and *D. melanogaster* PDF is proposed to be a functional homologue of VIP despite the lack of sequence similarity [28], [63]. Evolution of ligand-receptor systems is an enigma. In the case of some GPCRs co-evolution of metazoan ligand-receptor systems has occurred, while other structurally similar metazoan receptors are activated by unrelated peptides [31]. The divergent results within different GPCR groups raise intriguing questions about the evolution of ligand-receptor pairs and suggest that conservation of receptor function is not necessarily dependent on their activating molecule.

At present no ligands have been identified for the novel protostome cluster A and B receptors and deorphanization will be an essential step for characterization of their physiological function. *In silico* expression analysis revealed that the novel invertebrate cluster A receptors are principally distributed in insect Malpighian tubules and mid-gut suggesting that like the *D. melanogaster* DH44-R1, DH44-R2 and DH31-R they may also be involved in water excretion and diuresis (Table S8). The absence of representatives of cluster A and B in Diptera genomes is intriguing and may be an example of gene loss due to functional redundancy but studies are needed to test this hypothesis. The variable number of Class 2 B1 receptors identified in nematode and arthropod genomes and the existence of novel receptor clusters with unknown functions indicates that the physiological role of the invertebrate Class 2 B1 receptors remains at present incompletely characterized. While the metazoan receptors share common evolution as reflected by their sequence similarity and conserved gene environment, the evolution of their ligands is poorly explored [31], [56]. Evolutionary analysis of the vertebrate peptide ligands with the few identified in invertebrates suggests that they also emerged early and probably co-evolved with their receptors [31], [52]. However invertebrate receptor-peptide evolution is largely uncharacterized and the origin of the ligands in invertebrates remains to be described.

## Conclusion

Class 2 B1 GPCR ancestral-like subfamily genes are suggested to have emerged early, were present in the bilateral genome and underwent distinct evolutionary pressure after the protostome-deuterostome divergence. The distinct gene numbers for Class 2 B1 in the nematode and arthropod phyla and within members of the same phylum presumably results from species-specific gene/genome molecular evolution rates potentially favored by organismal generation time and adaption to their external environment. In vertebrates, the GPS-receptor clade and the CALCR and CRHRs clade of Class 2 B1 are suggested to have emerged from a common ancestral gene precursor prior to the deuterostome divergence [8], [13], [52]. Based on the results of the present study identifying novel nematode and arthropod receptor clusters an alternative evolutionary model is proposed for the Class 2 B1 receptors. We hypothesize that prior to the protostome–deuterostome divergence 4 putative ancestral Class 2 B1-like GPCR genes arose from duplication events in the bilateral genome and gave rise to the current complement of Class 2 B1 receptors through specific evolutionary pressures at work in the deuterostome and protostome lineages (Figure 7). The novel nematode and arthropod Class 2 B1 members identified in this study are orphans and the identification of their activating peptides will contribute to establish their evolution and function in metazoan physiology.

## Supporting Information

Figure S1 **Alignment of the concatenated transmembrane sequences of the deduced amino acid sequences of protostome Class 2 B1 receptor used for in silico and phylogenetic analyses.**
(PDF)

Figure S2 **Maximum likelihood phylogenetic tree of the nematode and arthropod Class 2 B1 receptors.** Reliability of internal branches is assessed using the bootstrapping method (100 bootstrap replicates). Analysis is based on the amino acid sequence alignment of the Class 2 B1 receptors TM regions and included 116 nematode and arthropod sequences.
(TIFF)

Figure S3 **Maximum likelihood phylogenetic tree of the nematode, arthropod and human Class 2 B1 receptors.** Reliability of internal branches is assessed using the bootstrapping method (100 bootstrap replicates). Analysis is based on the amino acid sequence alignment of the human Class 2 B1 receptors with the nematode and arthropod receptor genes possessing the seven TM regions.
(TIFF)

Figure S4 Gene environment comparisons of Class 2 B1 receptors in *C. elegans* (A) and *T. castaneum* (B) chromosomes. Gene symbols were obtained from ENSEMBL annotation and when not available for *T. castaneum* the homologue designation in *D. melanogaster* was used. Solid horizontal lines represent chromosome fragments and genes are represented with blocks. To facilitate visualization genes are colored according to which family they belong. The position of Class 2 B1 receptor genes within the *C. elegans* and *T. castaneum* chromosomes are annotated in color according to the phylogenetic analysis obtained from Figure 1. Lengths of the extracted chromosome regions that contain the Class 2 B1 GPCR genes are indicated (Mb). Only genes common between the species analyzed are represented.
(TIFF)

Figure S5 **Maximum likelihood phylogenetic tree of the nematode, arthropod and human Class 2 B1 receptors with the putative early deuterostome PDF-R-like receptors.** Reliability of internal branches is assessed using the bootstrapping method (100 bootstrap replicates). Analysis is based on the amino acid sequence alignment of the nematode and arthropod Class 2 B1 receptors with the seven TM regions.
(TIFF)

Table S1 **Accession numbers of Class 2 B1 receptor genes extracted from the nematode and arthropod genomes analyzed.** Nematodes are shaded. Genes annotated with an asterisk (*) indicate sequences that were not included in the invertebrate (blue, Figure 1) and invertebrate-human (red, Figure 2) phylogenetic tree analysis.
(PDF)

Table S2 **List of the accession numbers and adopted acronyms of the nematode and arthropod Class 2 B1 GPCR genes.** The symbols of the genes previously identified (*C. elegans*, *D. melanogaster* and *B. mori*) were maintained.
(PDF)

Table S3 **Percentage of amino acid sequence similarity of the nematode and arthropod cluster A members.** Comparisons were performed using at least 6 TM domains (Figure S1). Nematode sequences are shaded.
(PDF)

Table S4 **Percentage of amino acid sequence similarity of the nematode and arthropod cluster B members.** Only arthropod receptors with more than 6 TM identified were used with the exception of Tsp4 (marked with “*” in which 5 TM domains were considered (Figure S1). The nematode representative is shaded.
(PDF)

Table S5 **Amino acid sequence similarity of the** ***T. castaneum*** **Class 2 B1 receptors.**
(PDF)

Table S6 **Percentages of amino acid sequence similarity of the** ***C. elegans*** **and** ***T. castaneum*** **receptors with the human homologues and sea urchin (** ***Strongylocentroyus purpuratus*** **) PDF-R-like.** The *T. castaneum* cluster A member (Tca6) shares the lowest sequence similarity with the human receptors.
(PDF)

Table S7 **Gene list (accession number, chromosome position, symbol and initial gene position) of the nematode (** ***C. elegans*** **) and vertebrate human (** ***H. sapiens*** **) and chicken (** ***G. gallus*** **) gene sequence homologues of the** ***T. castaneum*** **Class 2 B1 gene environment on LG2, LG4, LG5 and LG9.** Data was obtained using the Ensembl Biomart software.
(PDF)

Table S8 **EST list and their tissue origin for the arthropod cluster A and cluster B Class 2 B1 receptor genes.** Searches were performed using the deduced amino acid sequence of the species-specific genes identified in the study and their identity confirmed against the species genomes.
(PDF)

File S1 **Sequences used to construct the Maximum likelihood phylogenetic tree of the nematode and arthropod Class 2 B1 receptors in FASTA format.**
(FASTA)

File S2 **Maximum likelihood phylogenetic tree of the nematode and arthropod Class 2 B1 receptors in Newick format.**
(NWK)

File S3 **Sequences used to construct the Maximum likelihood phylogenetic tree of the nematode, arthropod and human Class 2 B1 receptors in FASTA format.**
(NWK)

File S4 **Maximum likelihood phylogenetic tree of the nematode, arthropod and human Class 2 B1 receptors in Newick format.**
(NWK)

File S5 **Sequences used to construct the Maximum likelihood phylogenetic tree of the nematode, arthropod and human Class 2 B1 receptors with the putative early deuterostome PDF-R-like receptors in FASTA format.**
(NWK)

File S6 **Maximum likelihood phylogenetic tree of the nematode, arthropod and human Class 2 B1 receptors with the putative early deuterostome PDF-R-like receptors in Newick format.**
(NWK)

## References

[1] Fredriksson R, Lagerstrom MC, Lundin LG, Schioth HB (2003) The G-protein-coupled receptors in the human genome form five main families. Phylogenetic analysis, paralogon groups, and fingerprints. Mol Pharmacol 63: 1256–1272. 12761335 10.1124/mol.63.6.1256

[2] Schioth HB, Fredriksson R (2005) The GRAFS classification system of G-protein coupled receptors in comparative perspective. Gen Comp Endocrinol 142: 94–101. 15862553 10.1016/j.ygcen.2004.12.018

[3] Bayliss WM, Starling EH (1902) The mechanism of pancreatic secretion. J Physiol 28: 325–353. 16992627 10.1113/jphysiol.1902.sp000920 PMC1540572

[4] Dong M, Miller LJ (2002) Molecular pharmacology of the secretin receptor. Receptors Channels 8: 189–200. 12529936

[5] Ishihara T, Nakamura S, Kaziro Y, Takahashi T, Takahashi K, et al (1991) Molecular cloning and expression of a cDNA encoding the secretin receptor. EMBO J 10: 1635–1641. 1646711 10.1002/j.1460-2075.1991.tb07686.x PMC452832

[6] Segre GV, Goldring SR (1993) Receptors for secretin, calcitonin, parathyroid hormone (PTH)/PTH-related peptide, vasoactive intestinal peptide, glucagonlike peptide 1, growth hormone-releasing hormone, and glucagon belong to a newly discovered G-protein-linked receptor family. Trends Endocrinol Metab 4: 309–314. 18407176 10.1016/1043-2760(93)90071-l

[7] Donnelly D (1997) The arrangement of the transmembrane helices in the secretin receptor family of G-protein-coupled receptors. FEBS Lett 409: 431–436. 9224704 10.1016/s0014-5793(97)00546-2

[8] Harmar AJ (2001) Family-B G-protein-coupled receptors. Genome Biol 2: REVIEWS3013. 11790261 10.1186/gb-2001-2-12-reviews3013 PMC138994

[9] Bale TL, Vale WW (2004) CRF and CRF receptors: role in stress responsivity and other behaviors. Annu Rev Pharmacol Toxicol 44: 525–557. 14744257 10.1146/annurev.pharmtox.44.101802.121410

[10] Hoare SR (2005) Mechanisms of peptide and nonpeptide ligand binding to Class B G-protein-coupled receptors. Drug Discov Today 10: 417–427. 15808821 10.1016/S1359-6446(05)03370-2

[11] Neer RM, Arnaud CD, Zanchetta JR, Prince R, Gaich GA, et al (2001) Effect of parathyroid hormone (1–34) on fractures and bone mineral density in postmenopausal women with osteoporosis. N Engl J Med 344: 1434–1441. 11346808 10.1056/NEJM200105103441904

[12] Sherwood NM, Krueckl SL, McRory JE (2000) The origin and function of the pituitary adenylate cyclase-activating polypeptide (PACAP)/glucagon superfamily. Endocr Rev 21: 619–670. 11133067 10.1210/edrv.21.6.0414

[13] Cardoso JC, Pinto VC, Vieira FA, Clark MS, Power DM (2006) Evolution of secretin family GPCR members in the metazoa. BMC Evol Biol 6: 108. 17166275 10.1186/1471-2148-6-108 PMC1764030

[14] Fredriksson R, Schioth HB (2005) The repertoire of G-protein-coupled receptors in fully sequenced genomes. Mol Pharmacol 67: 1414–1425. 15687224 10.1124/mol.104.009001

[15] Ng SY, Chow BK, Kasamatsu J, Kasahara M, Lee LT (2012) Agnathan VIP, PACAP and their receptors: ancestral origins of today’s highly diversified forms. PLoS One 7: e44691. 22957100 10.1371/journal.pone.0044691 PMC3434177

[16] Pinheiro PL, Cardoso JC, Power DM, Canario AV (2012) Functional characterization and evolution of PTH/PTHrP receptors: insights from the chicken. BMC Evol Biol 12: 110. 22768871 10.1186/1471-2148-12-110 PMC3483286

[17] Couvineau A, Laburthe M (2012) The family B1 GPCR: structural aspects and interaction with accessory proteins. Curr Drug Targets 13: 103–115. 21777182 10.2174/138945012798868434

[18] Laburthe M, Couvineau A, Gaudin P, Maoret JJ, Rouyer-Fessard C, et al (1996) Receptors for VIP, PACAP, secretin, GRF, glucagon, GLP-1, and other members of their new family of G protein-linked receptors: structure-function relationship with special reference to the human VIP-1 receptor. Ann N Y Acad Sci 805: 94–109 discussion 110–101. 8993396 10.1111/j.1749-6632.1996.tb17476.x

[19] Parthier C, Kleinschmidt M, Neumann P, Rudolph R, Manhart S, et al (2007) Crystal structure of the incretin-bound extracellular domain of a G protein-coupled receptor. Proc Natl Acad Sci U S A 104: 13942–13947. 17715056 10.1073/pnas.0706404104 PMC1955799

[20] Nordstrom KJ, Lagerstrom MC, Waller LM, Fredriksson R, Schioth HB (2009) The Secretin GPCRs descended from the family of Adhesion GPCRs. Mol Biol Evol 26: 71–84. 18845549 10.1093/molbev/msn228

[21] Hector CE, Bretz CA, Zhao Y, Johnson EC (2009) Functional differences between two CRF-related diuretic hormone receptors in Drosophila. J Exp Biol 212: 3142–3147. 19749107 10.1242/jeb.033175

[22] Johnson EC, Bohn LM, Taghert PH (2004) Drosophila CG8422 encodes a functional diuretic hormone receptor. J Exp Biol 207: 743–748. 14747406 10.1242/jeb.00818

[23] Hauser F, Cazzamali G, Williamson M, Park Y, Li B, et al (2008) A genome-wide inventory of neurohormone GPCRs in the red flour beetle Tribolium castaneum. Front Neuroendocrinol 29: 142–165. 18054377 10.1016/j.yfrne.2007.10.003

[24] Hill CA, Fox AN, Pitts RJ, Kent LB, Tan PL, et al (2002) G protein-coupled receptors in Anopheles gambiae. Science 298: 176–178. 12364795 10.1126/science.1076196

[25] Zamanian M, Kimber MJ, McVeigh P, Carlson SA, Maule AG, et al (2011) The repertoire of G protein-coupled receptors in the human parasite Schistosoma mansoni and the model organism Schmidtea mediterranea. BMC Genomics 12: 596. 22145649 10.1186/1471-2164-12-596 PMC3261222

[26] Janssen T, Husson SJ, Lindemans M, Mertens I, Rademakers S, et al (2008) Functional characterization of three G protein-coupled receptors for pigment dispersing factors in Caenorhabditis elegans. J Biol Chem 283: 15241–15249. 18390545 10.1074/jbc.M709060200 PMC3258896

[27] Johnson EC, Shafer OT, Trigg JS, Park J (2005) Schooley DA, et al (2005) A novel diuretic hormone receptor in Drosophila: evidence for conservation of CGRP signaling. J Exp Biol 208: 1239–1246. 15781884 10.1242/jeb.01529

[28] Mertens I, Vandingenen A, Johnson EC, Shafer OT, Li W, et al (2005) PDF receptor signaling in Drosophila contributes to both circadian and geotactic behaviors. Neuron 48: 213–219. 16242402 10.1016/j.neuron.2005.09.009

[29] Li Y, Hoxha V, Lama C, Dinh BH, Vo CN, et al (2011) The hector G-protein coupled receptor is required in a subset of fruitless neurons for male courtship behavior. PLoS One 6: e28269. 22140564 10.1371/journal.pone.0028269 PMC3227663

[30] Li C, Chen M, Sang M, Liu X, Wu W, et al (2013) Comparative genomic analysis and evolution of family-B G protein-coupled receptors from six model insect species. Gene 519: 1–12. 23428791 10.1016/j.gene.2013.01.061

[31] Mirabeau O, Joly JS (2013) Molecular evolution of peptidergic signaling systems in bilaterians. Proc Natl Acad Sci U S A 110: E2028–2037. 23671109 10.1073/pnas.1219956110 PMC3670399

[32] Cardoso JC, Felix RC, Fonseca VG, Power DM (2012) Feeding and the rhodopsin family g-protein coupled receptors in nematodes and arthropods. Front Endocrinol (Lausanne) 3: 157. 23264768 10.3389/fendo.2012.00157 PMC3524798

[33] Saitou N, Nei M (1987) The neighbor-joining method: a new method for reconstructing phylogenetic trees. Mol Biol Evol 4: 406–425. 3447015 10.1093/oxfordjournals.molbev.a040454

[34] Felsenstein J (1985) Confidence Limits on Phylogenies: An Approach Using the Bootstrap. Evolution 39: 783–791. 28561359 10.1111/j.1558-5646.1985.tb00420.x

[35] Tamura K, Peterson D, Peterson N, Stecher G, Nei M, et al (2011) MEGA5: molecular evolutionary genetics analysis using maximum likelihood, evolutionary distance, and maximum parsimony methods. Mol Biol Evol 28: 2731–2739. 21546353 10.1093/molbev/msr121 PMC3203626

[36] Dereeper A, Guignon V, Blanc G, Audic S, Buffet S, et al (2008) Phylogeny.fr: robust phylogenetic analysis for the non-specialist. Nucleic Acids Res 36: W465–469. 18424797 10.1093/nar/gkn180 PMC2447785

[37] Thompson JD, Higgins DG, Gibson TJ (1994) CLUSTAL W: improving the sensitivity of progressive multiple sequence alignment through sequence weighting, position-specific gap penalties and weight matrix choice. Nucleic Acids Res 22: 4673–4680. 7984417 10.1093/nar/22.22.4673 PMC308517

[38] Asmann YW, Dong M, Ganguli S, Hadac EM, Miller LJ (2000) Structural insights into the amino-terminus of the secretin receptor: I. Status of cysteine and cystine residues. Mol Pharmacol 58: 911–919. 11040037 10.1124/mol.58.5.911

[39] Grace CR, Perrin MH, Gulyas J, Digruccio MR, Cantle JP, et al (2007) Structure of the N-terminal domain of a type B1 G protein-coupled receptor in complex with a peptide ligand. Proc Natl Acad Sci U S A 104: 4858–4863. 17360332 10.1073/pnas.0700682104 PMC1829229

[40] Pioszak AA, Xu HE (2008) Molecular recognition of parathyroid hormone by its G protein-coupled receptor. Proc Natl Acad Sci U S A 105: 5034–5039. 18375760 10.1073/pnas.0801027105 PMC2278174

[41] DeAlmeida VI, Mayo KE (1998) Identification of binding domains of the growth hormone-releasing hormone receptor by analysis of mutant and chimeric receptor proteins. Mol Endocrinol 12: 750–765. 9605937 10.1210/mend.12.5.0102

[42] Laburthe M, Couvineau A, Marie JC (2002) VPAC receptors for VIP and PACAP. Receptors Channels 8: 137–153. 12529932

[43] Richards S, Gibbs RA, Weinstock GM, Brown SJ, Denell R, et al (2008) The genome of the model beetle and pest Tribolium castaneum. Nature 452: 949–955. 18362917 10.1038/nature06784

[44] Savard J, Tautz D, Lercher MJ (2006) Genome-wide acceleration of protein evolution in flies (Diptera). BMC Evol Biol 6: 7. 16436210 10.1186/1471-2148-6-7 PMC1369000

[45] Wyder S, Kriventseva EV, Schroder R, Kadowaki T, Zdobnov EM (2007) Quantification of ortholog losses in insects and vertebrates. Genome Biol 8: R242. 18021399 10.1186/gb-2007-8-11-r242 PMC2258195

[46] Coghlan A (2005) Nematode genome evolution. WormBook: 1–15. 10.1895/wormbook.1.15.1 PMC4781476 18050393

[47] Witherspoon DJ, Robertson HM (2003) Neutral evolution of ten types of mariner transposons in the genomes of Caenorhabditis elegans and Caenorhabditis briggsae. J Mol Evol 56: 751–769. 12911038 10.1007/s00239-002-2450-x

[48] Mitreva M, Jasmer DP, Zarlenga DS, Wang Z, Abubucker S, et al (2011) The draft genome of the parasitic nematode Trichinella spiralis. Nat Genet 43: 228–235. 21336279 10.1038/ng.769 PMC3057868

[49] Sommer RJ, Streit A (2011) Comparative genetics and genomics of nematodes: genome structure, development, and lifestyle. Annu Rev Genet 45: 1–20. 21721943 10.1146/annurev-genet-110410-132417

[50] Simakov O, Marletaz F, Cho SJ, Edsinger-Gonzales E, Havlak P, et al (2013) Insights into bilaterian evolution from three spiralian genomes. Nature 493: 526–531. 23254933 10.1038/nature11696 PMC4085046

[51] Engsontia P, Sanderson AP, Cobb M, Walden KK, Robertson HM, et al (2008) The red flour beetle’s large nose: an expanded odorant receptor gene family in Tribolium castaneum. Insect Biochem Mol Biol 38: 387–397. 18342245 10.1016/j.ibmb.2007.10.005

[52] Hwang JI, Moon MJ, Park S, Kim DK, Cho EB, et al.. (2013) Expansion of Secretin-Like G Protein-Coupled Receptors and Their Peptide Ligands via Local Duplications Before and After Two Rounds of Whole-Genome Duplication. Mol Biol Evol. 10.1093/molbev/mst031 23427277

[53] Meelkop E, Temmerman L, Schoofs L, Janssen T (2011) Signalling through pigment dispersing hormone-like peptides in invertebrates. Prog Neurobiol 93: 125–147. 21040756 10.1016/j.pneurobio.2010.10.004

[54] Cabrero P, Radford JC, Broderick KE, Costes L, Veenstra JA, et al (2002) The Dh gene of Drosophila melanogaster encodes a diuretic peptide that acts through cyclic AMP. J Exp Biol 205: 3799–3807. 12432004 10.1242/jeb.205.24.3799

[55] Furuya K, Milchak RJ, Schegg KM, Zhang J, Tobe SS, et al (2000) Cockroach diuretic hormones: characterization of a calcitonin-like peptide in insects. Proc Natl Acad Sci U S A 97: 6469–6474. 10841553 10.1073/pnas.97.12.6469 PMC18626

[56] Cardoso JC, Vieira FA, Gomes AS, Power DM (2010) The serendipitous origin of chordate secretin peptide family members. BMC Evol Biol 10: 135. 20459630 10.1186/1471-2148-10-135 PMC2880984

[57] Coast GM, Orchard I, Phillips JE (2002) Schooley DA (2002) Insect diuretic and antidiuretic hormones. Advances in Insect Physiology 29: 279–409.

[58] Skaer NJ, Nassel DR, Maddrell SH, Tublitz NJ (2002) Neurochemical fine tuning of a peripheral tissue: peptidergic and aminergic regulation of fluid secretion by Malpighian tubules in the tobacco hawkmoth M. sexta. J Exp Biol 205: 1869–1880. 12077163 10.1242/jeb.205.13.1869

[59] Taghert PH, Veenstra JA (2003) Drosophila neuropeptide signaling. Adv Genet 49: 1–65. 12779250 10.1016/s0065-2660(03)01001-0

[60] Zandawala M (2012) Calcitonin-like diuretic hormones in insects. Insect Biochem Mol Biol 42: 816–825. 22820711 10.1016/j.ibmb.2012.06.006

[61] Andreotti G, Mendez BL, Amodeo P, Morelli MA, Nakamuta H, et al (2006) Structural determinants of salmon calcitonin bioactivity: the role of the Leu-based amphipathic alpha-helix. J Biol Chem 281: 24193–24203. 16766525 10.1074/jbc.M603528200

[62] Nery LE, Castrucci AM (1997) Pigment cell signalling for physiological color change. Comp Biochem Physiol A Physiol 118: 1135–1144. 9505423 10.1016/s0300-9629(97)00045-5

[63] Mertens I, Husson SJ, Janssen T, Lindemans M, Schoofs L (2007) PACAP and PDF signaling in the regulation of mammalian and insect circadian rhythms. Peptides 28: 1775–1783. 17586087 10.1016/j.peptides.2007.05.005

[64] Rota-Stabelli O, Daley AC, Pisani D (2013) Molecular timetrees reveal a cambrian colonization of land and a new scenario for ecdysozoan evolution. Curr Biol 23: 392–398. 23375891 10.1016/j.cub.2013.01.026

[65] You M, Yue Z, He W, Yang X, Yang G, et al (2013) A heterozygous moth genome provides insights into herbivory and detoxification. Nat Genet 45: 220–225. 23313953 10.1038/ng.2524

[66] Hedges SB, Blair JE, Venturi ML, Shoe JL (2004) A molecular timescale of eukaryote evolution and the rise of complex multicellular life. BMC Evol Biol 4: 2. 15005799 10.1186/1471-2148-4-2 PMC341452

[67] Frooninckx L, Van Rompay L, Temmerman L, Van Sinay E, Beets I, et al (2012) Neuropeptide GPCRs in C. elegans. Front Endocrinol (Lausanne) 3: 167. 23267347 10.3389/fendo.2012.00167 PMC3527849

[68] Meelkop E, Temmerman L, Janssen T, Suetens N, Beets I, et al (2012) PDF receptor signaling in Caenorhabditis elegans modulates locomotion and egg-laying. Mol Cell Endocrinol 361: 232–240. 22579613 10.1016/j.mce.2012.05.001

[69] Jee C, Lee J, Lim JP, Parry D, Messing RO, et al (2013) SEB-3, a CRF receptor-like GPCR, regulates locomotor activity states, stress responses and ethanol tolerance in Caenorhabditis elegans. Genes Brain Behav 12: 250–262. 22853648 10.1111/j.1601-183X.2012.00829.x PMC3848202

[70] Renn SC, Park JH, Rosbash M, Hall JC, Taghert PH (1999) A pdf neuropeptide gene mutation and ablation of PDF neurons each cause severe abnormalities of behavioral circadian rhythms in Drosophila. Cell 99: 791–802. 10619432 10.1016/s0092-8674(00)81676-1

[71] Talsma AD, Christov CP, Terriente-Felix A, Linneweber GA, Perea D, et al (2012) Remote control of renal physiology by the intestinal neuropeptide pigment-dispersing factor in Drosophila. Proc Natl Acad Sci U S A 109: 12177–12182. 22778427 10.1073/pnas.1200247109 PMC3409758

[72] Veenstra JA, Agricola HJ, Sellami A (2008) Regulatory peptides in fruit fly midgut. Cell Tissue Res 334: 499–516. 18972134 10.1007/s00441-008-0708-3

[73] Kokkoris S, Andrews P, Webb DJ (2012) Role of calcitonin gene-related peptide in cerebral vasospasm, and as a therapeutic approach to subarachnoid hemorrhage. Front Endocrinol (Lausanne) 3: 135. 23162536 10.3389/fendo.2012.00135 PMC3498620

[74] Naot D, Cornish J (2008) The role of peptides and receptors of the calcitonin family in the regulation of bone metabolism. Bone 43: 813–818. 18687416 10.1016/j.bone.2008.07.003

[75] Janssen D, Kozicz T (2013) Is it really a matter of simple dualism? Corticotropin-releasing factor receptors in body and mental health. Front Endocrinol (Lausanne) 4: 28. 23487366 10.3389/fendo.2013.00028 PMC3594922

[76] Lewis K, Li C, Perrin MH, Blount A, Kunitake K, et al (2001) Identification of urocortin III, an additional member of the corticotropin-releasing factor (CRF) family with high affinity for the CRF2 receptor. Proc Natl Acad Sci U S A 98: 7570–7575. 11416224 10.1073/pnas.121165198 PMC34709

[77] Perrin MH, Vale WW (1999) Corticotropin releasing factor receptors and their ligand family. Ann N Y Acad Sci 885: 312–328. 10816663 10.1111/j.1749-6632.1999.tb08687.x

[78] Dobolyi A, Dimitrov E, Palkovits M, Usdin TB (2012) The neuroendocrine functions of the parathyroid hormone 2 receptor. Front Endocrinol (Lausanne) 3: 121. 23060860 10.3389/fendo.2012.00121 PMC3465808

[79] Gensure RC, Gardella TJ, Juppner H (2005) Parathyroid hormone and parathyroid hormone-related peptide, and their receptors. Biochem Biophys Res Commun 328: 666–678. 15694400 10.1016/j.bbrc.2004.11.069

[80] Drozdowski L, Thomson AB (2009) Intestinal hormones and growth factors: effects on the small intestine. World J Gastroenterol 15: 385–406. 19152442 10.3748/wjg.15.385 PMC2653359

[81] Kim W, Egan JM (2008) The role of incretins in glucose homeostasis and diabetes treatment. Pharmacol Rev 60: 470–512. 19074620 10.1124/pr.108.000604 PMC2696340

[82] Korner M, Christ E, Wild D, Reubi JC (2012) Glucagon-like peptide-1 receptor overexpression in cancer and its impact on clinical applications. Front Endocrinol (Lausanne) 3: 158. 23230431 10.3389/fendo.2012.00158 PMC3515855

[83] Dickson L, Finlayson K (2009) VPAC and PAC receptors: From ligands to function. Pharmacol Ther 121: 294–316. 19109992 10.1016/j.pharmthera.2008.11.006

[84] Laburthe M, Couvineau A, Tan V (2007) Class II G protein-coupled receptors for VIP and PACAP: structure, models of activation and pharmacology. Peptides 28: 1631–1639. 17574305 10.1016/j.peptides.2007.04.026

[85] Lam IP, Siu FK, Chu JY, Chow BK (2008) Multiple actions of secretin in the human body. Int Rev Cytol 265: 159–190. 18275888 10.1016/S0074-7696(07)65004-9

[86] Vaudry D, Falluel-Morel A, Bourgault S, Basille M, Burel D, et al (2009) Pituitary adenylate cyclase-activating polypeptide and its receptors: 20 years after the discovery. Pharmacol Rev 61: 283–357. 19805477 10.1124/pr.109.001370

