# Supplementary material for: Nematode and Arthropod Genomes Provide New Insights into the Evolution of Class 2 B1 GPCRs
Source: PLoS One. 2014 Mar 20;9(3):e92220. doi: 10.1371/journal.pone.0092220 (PMC3961327; doi:10.1371/journal.pone.0092220)
Supplement: Figure S1 — Alignment of the concatenated transmembrane sequences of the deduced amino acid sequences of protostome Class 2 B1 receptor used for in silico and phylogenetic analyses. (PDF) [file pone.0092220.s001.pdf]

Figure S1

TM1 TM2 TM3 TM4 TM5 TM6 TM7

DH44-R receptors

```
Dme_DH44R1          : IIYYIGYTLSLVSLSLALIVFAYFKTIHANLFFTYIMSALFWILLLSVQIALITLFHFFTLTNFFWMLVEGLYLFNIYASIGWGGPALFVVTWAVAKSLTWIYQGPVCAVLIINLTFLLRIMWVLIAAKALLVLIPLFGITYLVVLAAVLRAVLLSTQGFSVSLFYCFL
Dme_DH44R2          : IIYAGGYFLSFATLVVALIIFLSFKTIHANLFLTYITSALLWILTLFLQVTLVIMFQYFYLTNFFWMFVEGLYLFIIYALIGWGCPAVCILVWSIAKAFAWIFKVPASLALLVNLVFLIRIMWVLIASKALLVLIPLFGITYLLVLTEAIRAFLISTQGFFVALFYCFL
Aae_AAEL008292      : VIYFVGYTISLLALFCAVTVLVYFKTIHVNLFVTYIMSSSLWIIILSQQLFLVTLFHYFSTTNFFWMLVEGLYLFWKYSIIGWGGPLIFVGAWAIAKSFYWIIQGPSCAVLVINLIFLLRIMWVLIASKALLVLIPLLGITYLVVIYAVTRAVLLSTQGLVVSLLYCFL
Aae_AAEL8287_5894   : IIYYSGYIVSLVALSLAVIVFVYFKTIHANLFITYILSALMWIIILTLQLILVTLLHYFTLTNFFWMLVEGLYLFNMYAAIGWG----------------WIFQGPVCAVLIINLVFLIRIMWVLIASKALLVLIPLLGITYLVVLAAVARALLLSTQGFSVSLFYCFL
Aga_AGAP005464      : LIYFVGYSISLAALVLAVAVLVYFKTIHVNLFLTYIMSSSLWILILSLQIFLVTLFHYFSTTNFFWMLVEGLYLFRKYAIIGWGGPLIFVGAWAIAKPFFWIIQGPSCAVLVINLIFLLRIMWVLIASKALLVLIPLLGITYLIVIYAITRAILLSTQGFVVSLLYCFL
Aga_AGAP005465      : IIYYTGYILSLVALSLAVIVFVYFKTIHANLFITYILSALLWIIILTLQLIFVTLLHYFTLTNFFWMLVEGLYLFNMYAAIGWGGPAICVILWAIAKGITWIFQGPVCAVLIINLVFLIRIMWVLIASKALLVLIPLLGITYLVVLAAIARALLLSTQGLSVSLFYCFL
Ada_ADAR2980_9352   : IIYYTGYTLSLVALSLAVIVFVYFKMSSS-----------LWILILSLQIVLVTLFHYFSATNFFWMLVEGLYLFRKYAIIGWGGPLIFVGAWAIAKPFFWIIQGPTCAVLAINLIFLLRIMWVLIASKALLVLIPLLGITYLIVIYATTRALLLSTQGFVVSLLYCFL
Cqu_CPIJ8821_8820   : IIYYSGYIISLIALSLAVIVFVYFKTIHANLFITYILSALMWMVILTQQLIFVTLLHYFTLTNFFWMLVEGLYLFNMYAAIGYG----------------WIFQGPVCAVLIINLVFLVRIMWVLIASKALLVLIPLLGITYLVVLAAVARALLLSTQ-----------
Cqu_CPIJ008822      : ------------------------------------------------------------------------------------GPVIFVGAWALTRPFYWIIQGPTCTVLVINLIFLLRIMWVLIASKALLVLIPLLGITYLIVIYAVTRAILLSTQGFVVSLLYCFL
Ame_GB10976         : TIYFAGYSISLITLIIAVSIFLYCKNIHTNLMFTYILADFMWILNNVMQVAFLSLFHYFQLTNYFWMFVEGLYLLRLCLIIGWGIPVLFITMWCIAKSLDWFYQAPAILVLCVNVVFLFMIMWVLIASKALLVLIPLLGVTYVLVLTSYARAVLLSSQGLFVALFYCFL
Nvi_NV11249         : NLYLIGYGLSLSTLIIAVIIYLYYRIIHTNLMFTYILANFLWILMIVSQVIFYSMYHYFQLTNYFWMFVEGLYLLKTCLFIGWGIPLIVVLVWGIIKALAWLYQVPAILVLCVNVIFLFMIMWVLIAAKALLVLIPLLGVTYILMIAAYSRAVLLSSQGLLVALFYCFL
Ace_ACEP12831       : TLYFIGYSLSLFTLIMAVCIFIYYK------------------------------------------------------------------------------------------------------------------------------------------------
Tca_TC007104        : TIYFIGYTVSLVALLFAVYIFWKFKTIHMNLMCSYILADFMWIFVYSLQVFLIILLHYFHLTNFFWMFVEGLYLPRIYAVIGWGGPILFVLVWGIAKSFTWIYQGPAIAVLIINVIFLCIIMWVLIAAKALLVLIPLLGVTYILVIVDSIRAILLSTQGFTVALFYCFL
TcaTC0127999        : TIYFVGYVLSLITLSIALGIFTYFKRIHMNLMWSYMLMYIMWILTLTVLGFVITLLHYFHISTFFWMFVEGLYLLRVYVCIGWGLPMIFILVWVIVKSFIWIFQGPTMLVLLLNLAFLLAIMWVLIAAKALLVLMPLLGITYVITIYECVRAVLLSTQPL---------
Bmo_BGIBMGA001910   : ------------------------------------------------------------------------------------------------------HKAPALAGLALNLFFLVRIMWVLATK-ALLVLIPLLGITNLLVLCDYARALMLSTQGFTVALFYCFM
Dpl_KGM20522        : LIYLAGYTLSLAVLSLAVFVFLYFKTIHTNLMSTYILSACSWMLNLALQNILVICMHYFYLTNFFWMLVEGLYLLKVYTTIGWGAPAIFITIWVIARCFVWIHKAPALVGLALNLFFLIRIMWVLIATKALLVLIPLLGITNLLVLCDYTRAVMLSTQVCIELMKEIKN
Hme_HMEL014214      : --------------------------------------------------ILVICMHYFYLTNFFWMLVE--------------------------VPSTWIHKAPALAGLALNLFFLIRIMWVSGAG-AVRAERRLV--------------------GFTVALFYCFM
Hme_HMEL002706      : LIYLAGYSLSLGVLSLAVFVFLYFKTIHTNLMSTYILSACSWMLNLALQNILVICMHYFYLTNFFWMLVEARGG-----------------------------------------------------------------------------------------------
Api_ACYPI00733      : YLYIAGHCLSLIMTSLAIFVFCRFKKIHSNLMASYLLAGIMWILNYTNLTLLVLPLYYFTMTNYFWAFIEGIPL-----------------------------------------------------------------------------------------------
Api_ACYPI54924      : YFYYGGYTISLVALVAAVSIFVYFKTIHTNLMCTYILSDFTWILTSTLQELFTFSLHYFVLTNFFWMFVEGLYLLRVYMFIGWGFPLVIMIVWGVSKIITWIYQGPAIIVLIVNLVFLSKIMWVLIASKALLVLIPLLGVTYILTMVSYGRATLLSMQGFMVAIFYCFV
Rpr_RPRC000578      : TIYAVGYGFSLTALGLAVWIFLYYKTIHTNLMCTYILADLMWILSSIQVYVLFILLHYLILTNYFWMFVEGLYLLRAYLAIGWGIPVIIVIPSCLARAFIWIYMTSSIIVLAVNVIFLIMIMWVLIATKALLVLIPLLGVTYILFIASYIRAFLLSTQGLMVALLYCFL
Phu_PHUM132710      : LLYYIGYSVSVVALLIAMFIFLYFKTIHTNLMFTYVMADFMWILTGILQNMLPLVLHYFHVTTFFWMFVEGLYLTGVYVFIGWGIPLILIIIWGIVKAVAWIFKIPVLTVMALNSVFLVMIMWVLIATKALLVLIPLLGITYILTIVYNMRAFMLSFQGFLVALFYCFL
Dpu_DappuP62157     : TIYFTGYTVSIVALTLAIWIFIHFKTIHTHLLITYVMADLLWIVTATLQLIIVIFLHYFHLTNFFWMFVEGFYLLRIYALIGWGIPLPVVIVWAVVKANWWIYMTPAIVVLFVNILFLVKIMWVLIGSKALLVLIPLLGLTYVLVIASHIRAVLLSTQGFLVTLFFCFL
Dpu_DappuP58251     : TIYFTGYTVSIVALTLAIWIFIHFKTIHTHLLITYIMADLLWIVTATLQLIIVIFLHYFHLTNFFWMFVEGFYLLRIYAFIGWGIPLPVVIVWAVVKDLEWIYMTPAIVVLFVNILFLVKIMWVLIGSKALLVLIPLLGLTYVLVIASHIRAVLLSTQGFLVTLFFCFL
Isc_ISCW019312      : TLYYIGYVLSLFALSVALWIFLYYKTIHINLMITYFLISLTWIVTATLQSFLYIVLTYLMGTNFFWMFVEGLYLICLFIGW----------------------------GLPAIIICTWATTKVYLAAKALLVLIPLLGVTYILVIW-YLQATLLSTQGFTVAVLYCFL
Isc_ISCW007036      : MLCYIGYSLSFAALTVAIWIFSFYRTIHVNLMVTYLLISITWIITASLQSFLYIVLTYLMGTNFFWMFVEGLYLMHVYVFIGWGI---------------CIFIAPVMLVLLMNIIFLGGIMWVLIAAKALLVLIPLLGVTYILVIWTYLQVALLPTQGFTVAVLYCFM
Isc_ISCW007612      : TIYYIGYGMSLCALTIALWIFLYYKTIHVNLMVTYFLISITWITTATLQSFMYVVLTYLMGTNFFWMFVEGLYLMHVYAFIGWGIPAVVVTIWAVTK-AYCVFIVPVILVLLTNIFFLSQIMWVLIAAKALLVLIPLLGVSYILVIWTYVQITLFSTQGFTVAVLYCFL
Isc_ISCW019068      : ----------------------------------------------------------------------------------------------------CIFICPVVIVLLVNIFFMGEIMWVLIAAKALLVLIPLLGVTYILVIWTYLQITLLSTQ-----------
Tur_tetur01g00270   : TIYYFGYGLSLLALTTALFIFLYFKTIHTHLIVTYELFALTWIITATLQSFLYILLPYFTGTQFFWMFIEGLYLIHFYATIGWGLPALVVLFWAPSKYFSSIFICPILFVLMLNIFFLCKIMWVLIAAKALLVLTPLLGVTYILFLVTYLQATLYSTQGFTVSVFYCFL
Tur_tetur01g03970   : IVYYFGYGLSIVALFIALAILITFKTIHINLMLSYMFHALTWIATASLQDFLYILLTYFTASSFFWMFLEGLYLLILFALIGWGFPVILVTLWAPIKYLYLIYICPISIVLVVNLFFLGVIIYVLVAVKALLVLTPLLGIAYILLLVTYIHATLNSTQGFTVAVLYCFL
```

TM1 TM2 TM3 TM4 TM5 TM6 TM7

DH31-R/Hec-R receptors

```
Dme_DH31R1         : LISEVGYGTSLLAILLSLAILGYFKTLHMNLFASFAANNSLWLVWYLLVMALHITLHYFLLSNYSWMLCEGFYLVKWLIAFGWGSPAIVIFVYSMARGLGNILMVPVCISMFLNLLFLCNIVRVVLAFRATLLLVPLLGLQYILTPFEIISAFTASFQGLCVAILFCFC
Aae_AAEL010043     : ----------------------------------------------------TLHLVLHYFLITNYAWMLCEGFYLVNWLVVLGWTTPGIVIMAYGFLRGYANVFKAPVCISMLLNLLFLCNIIRVVLAFRATLLLVPLLGLQYILTPFEIISAFTASFQGLFVAVLFCFF
Aga_AGAP009770     : LIYETGYAISLIAILLSLAILSYFRTLHMNLFVSFACNNSLWLLWYRLVLTLHLVLHYFLLTNYSWMLCEGFYLLKWLLALGWGSPAFFIVLYGFLRGYAKVFVGPVCISMLLNLVFLFNIMRVLLAFRATLLLVPLLGLQYILTPFETMLACTSSFQGLFVAVLFCFL
Ada_ADAR009785     : LIYETGYCISLIAILLSLAILSYFRTLHMNLFASFACNNFLWLLWYRLVLTLHLVLHYFLLSNYSWMLCEGFYLLKWLLALGWGSPIFFIVLYGFLRGY-KVFIVPVCSSMLLNLVFLFNIMRVLLAFRATLLLVPLLGLQYILTPFETILASTSSFQGLFVAILFCFL
Cqu_CPIJ014419     : TIYETGYSISLIALILSLGILSYFRTLHMNLFASFATNNTLWLLWYRMVLTLHLVLHYFLITNYAWMLCEGFYLVKWLVVLGWTVPACVIVLYGVLRGTYMVFIVPVCISMLLNLLFLCNIVRVVLAFRATLLLVPLLGLQYMLTPFETISAFTASFQGLFVAVLFCFF
Ame_GB12975        : GLYEAGYAISLIALLLSLGILTYFRTLHMNLFASFAVNNALWLVWYRCIVLLHIVLHYFLLTNYAWMLCEGFYLVKWLMILGWPVPAIIVTIYACLRATSNVLVYPVCVSTLLNVLFLFNIVRVLLAFRATLLLVPLLGLHYLVIPFEVLSAITASFQGLCVAILFCFC
Nvi_NV14697        : VLYETGYTISLVALLVSLGILTYFRTLHMNLFASFAANNALWLIWYGSVVFLHVILHYFLLTNYAWMLCEGFYLVKWLMALGWPIPAIIVTIYTVLRATSVVLIYPVCVSTLLNLLFLCNIVRVLLAFRATLLLVPLLGLHYLVIPFGVMSAITASFQGLCVAILFCFC
Ace_ACEP13106      : GIYEVGYAISLVALLLSLGILTYFRTLHMNLFTSFAFNNALWLIWYRFIVVLHVVLHYFLLTNYAWMLCEGFYLVNWLMGLGWPVPAVIVTLYTALRASS---------------------------------------------------------------------
Tca_TC002694       : LIYCVGYGVSLVALLVSLALLTYFKTVHMNLFSSFAMNNFLWLLWYSLVVVLHVVLFTFLISNYSWMLCEGIYLLRCMLALGWGIPLLTTSIYAPVRSVLKILMVPVVITVFLNVIFLVNIVRVLLALRATMLLVPLLGLNFLLTPFEVVSALTASLQGIQPFLMFFWY
Bmo_BNGRB1         : VVYEAGYSVSLVALLLSLAILLYFRTVHMNLFGSFAVNNALWLAWYGLVVALNAVLQYAMLTNYMWMLCEGMYLVRALVAAGWMLPLPCILIYATRRALDPELAVPVGLAVLLNLCFLCNTVRVLCALRATCLLAPLLGLQYLLMPFEYATAAATSLQGLCVAVLYCFC
Dpl_KGM05922       : AVYETGYSVSLVALLVSLGILSYFKTVHMNLFTSFAFNNALWLAWYALVVLLNSTLQYALLTNYTWMLCEGLYLVRSLLAAGWLLPVLPAVIHAARRA--PELAVLVCVAVLLNLAFLCNIVRVLCALRATCLLAPLLGLQYLLTPFEYLSALTTSLQGLCVAVLYCFC
Hme_HMEL004721     : AVYETGYSVSLVALLLSLRCARITVTVHINLFASFALNNALWLAWYGLVVALNAALQYVLLTNYTWMLCEGLYLVRALLAAGWLLPVPSALVNAARRG--PELAVLVCLAVLLNLAFLCNIVRVLCALRATCLLAPLLGLQYLLTPFEYVSALTTSLQGLCVAILYCFC
Api_ACYPI007222    : --------------------------TLHTHMFTSFAINNLLWLLWYRLVVILHVILHYFLLTNYAWMLAEGFYLVRWSWTLAWSTPLVVISLYTLLRTIYEVLVVPVCMSMALNLVFLCNIVRVLWAFRATLLLLPLLGLHYLLTPFEVVSATTSSFQGLCVATLFCFL
Api_ACYPI001361    : MIYKWGYTVSLAALAVSIFIFFYFRQIHKNLFISLAVNNCLWLVWYEAVVLLHVLVQYFLVATYFWMFCEGLYLMPFLHTIGWGIPALLVSTYAALRTATWILSGPVCLSMLANLVFLINIVRLLLAVRATLILIPLLGLQYIVTPFQVTSAVVASCQG----------
Rpr_RPRC009814     : NIYQAGYFISLLALLLSLFILSYFKTLHMNLFTAFAFNNFLWLLWYRLVICLHVILHYFLLSCYAWMLAEGVYLVKVLTVLSWFVPIVFITLYTTLRLASTVLIILVATSMGLNFIFLCNIMRVVVALRATLLLLPLLGLNYLLTPFELISAVTASFQGLCVATLFCFC
Rpr_RPRC004735     : MIYKGGYAISLAALTLSIFIFFYFKQIHKSLFISLAVNNLLWLIWYEAVALLHILVQYFLVATYLWMFCEGLYLMPFLHLIGWGVPAILVTIYAILRMSNWTLSGPVLVSMIANFVFLINIVRLLLAVRATLILIPLLGLQYIVTPFQVTSALVASCQGLCVALLFCFC
Phu_PHUM428070     : QIYEIGYLISLIALLASLAIYTYFKTLHIHLFVSFAVNNALWLIWYRFILIFHVVLHYFLMTNYSWMLCEGLYLVRWLFVLGWAIPGIAIIVYLSLRANGNVLVVPVFISMVLNLVFLCNIVRVLMAFRATLLLVPLLGLHYLLTPFEVLSAITASLQGLCVATLFCFC
Dpu_DappuP62111    : NVYIAGYSVSLIALVMSLAILIAFRYIHRNLFASFIINNAMWLLWYKCVAILHVLLHYFLVSNYFWMFCEGLYLMKWFHLIGWALPAVVTSIYAGVRA--WILSGPVCLSMLTNAFFLVNIVRVLVNCKATLILLPLLGLHYVVMPFQIVSAIFTSFQGLCVAFLFCFC
Isc_ISCW012970     : SLYVGGYSISLVALLLSLFIFFYFRTIHKNLFTSFIINNLCWILWYIHVIVLHVVTQYFLLCNYLWMFCEGLYLLKWFLLIGWGFPLLPAIGYGVARRMD---------------------------------------------------------------------
Tur_tetur15g02300  : NLYIIGYSISVLALIISLIIFLSFKTIHKNLFISFIINNLMWIMWYTLVVMLHVVVNYFLLANYSWTFCEGLYLMKWFYVIGWGIPLIFIGFYAGFRGLSWWYTGPVLVSFVLNLFFLVNIVRVLVAVKATLILLPLLGLHFLVTPFEIVAALVASLQGLCVAILFCFC
Dme_HecR           : ELYVKGYALSLLALLISIIIFLGFKRIHVHLFASLACTCVAWILWYRLVVGLHLVVHYFMLVNYFWMFCEGLHLMRWFIVISWFSPIPIAIVYGLARHFSWIFSVPITLSLLASFIFLINVLRVIVAVRATIILVPLFGLQHFLLPYQMLSVVLVSLQGFVVSFLFCFA
Aae_AAEL006490     : DLYIGGYTISLVTLIVSLCDSEVHS---------------------------ALHILLHYLMLVNYFWMFCEG-----------------------------WLLTIPVCFSLVASLVFLINVVRVLLATRATLILIPLFGLQHILLPFQVVSAVLISLQGACVSCLFCFA
Aga_AGAP001175     : DIYIGGYTVSFLTLIISLCIFHSFRRIHIHLFTSLALSCLFWIVWYKFVVGLHILLHYLMLVNYFWMFCEGLHLMRWFVTIGWILPMVLVSLYALVRNTQWLLTIPVCFSLVASLVFLINVVRVLLATRATLILIPLFGLQHILLPFQIGAALLISLQGLCVSCLFCFA
Cqu_CPIJ011559     : DLYIGGYTISLVTLIVSLCVFFSFRRIHINLFISLALSCIFWLVWYKLVVSLHILLHYLMLVNYFWMFCEGLHLMRWFMTIGWIVPMGLISFYATFRNNYWLLTVPVFLSLAASLVFLVNVVRVLLATRATLILV----------------------------------
Tca_TC013321       : HLFVIGYSISLAALVISLAIFFTFRRIHIQLFISFALNNLMWIIWYKEVVALHLVVHYLMLANYMWMFCEGLHLMKWFFALGWGAPFIIVLIYSVVRIFIWILTAPVCISLLVSLIFLINVLRVILAARAALILIPLFGLQHILIPFQYVTVVVVTLQGLCVSCLFCFA
Bmo_BNGRB4         : NMYVGGYAISLIALILSLMIFVFLRRIHANLFISFILNNIMWIVWYKTVVSLHILTYYHMMTSYMWMFCEGLHLVRSYCAIGWAIPALVVALYTGVRMQLWIIVIAVVIILTLSFLFLVNVIRVLLAARATYFMIPLYGLHFILIPFQVVSALLTSLQGLCVSILFCFT
Dpl_KGM02552       : EIYVGGYSISVAALILSLFIFFYFKRVHMNLFTAFALNNILWIVWYRVVVLLHIVTTYFMVTGYTWMFCEGLHLMRCFLAIGWGLSLVIIIVYAVVRYHIWIITVPVVISLIASFVFLINVVRVLLAARAALILIPLFGLHFVLIPFQVVSALLTSLQGVCVAILFCFT
Api_ACYPI009569    : ----------------------------------------------------SLHVALQFFMVANYMWMFCEGLHLMRWFYAIGWGVPVALTVLYVSWRDISWVLTVPVFASILTSLMFLMNVMRVLLAVRAALILVPLFGIHYILIPYQIFSVVLVSTQGLCVSVLFCFA
Rpr_RPRC004753     : TIYVVGYYISFAALVLSLIIFLMFRAIHVQLFSSFAANNLMWIIWYKTVVVLHVILQYFMVANYLWMFCEGLHLMRCIIVLSFRCSIIIRSKFPNSKTPKWILIVPVCLSLFASLGFLINVVRVLLAVRAALILVPLFGIHHILIPFEIFSALLVSLQGFCVSVLFCFV
```

TM1 TM2 TM3 TM4 TM5 TM6 TM7

PDF-R and PDF-R related receptors

```
Cel_Pdf1                : KLEFVGLGLSLVSLILAISIFSYFRLLHLHLMIAMLMVVILRLVLYIDLIGMFFFLEYFKTVTFCWMFLEGIYLLLPYFIAGYGIPLVHTMLWLLVVLIKWILDGPRMAELVINLFFICNVIRVLYSVKAAMMLLPLLGVPNIMQTITYTASFTYMYQGLMVASIYCFT
Cel_Seb-3               : SVSLTLSFVSVILLVAAIVLFSIFDSIHKNLATAFVFRFAVLAIWTIVQTAILWFVIYFNVASVMWMLIEGAFLWSLYLACGWGVPFVVVTAWALVHQYIWILAGTMGSALIMNLIFLLMIVVILVTIKATLLLVPLLGISNIPLFYMLGSAILQHSQGIFIAVLYCFL
Hco_Hc_scaffold0394     : -----DEVIYIMNNLNNESLGVYFRMLHLHLMIAILMVVIIRLVLYIDLIAMYFLLEYFKTVAFGWMFLEGFYLLTPYLIAGYGGIPLLHTLIWLFVVLIWILDGPRMAELVVSVNLFFICNVIRV---AALMLIPLLGIPNIMQTITYLASFTYMYQGLMITIIYCFT
Hco_Hc_scaffold1996     : SVSLTLSIVSVALLVAAILLFSIFDSIHKNLATAFVFRFAVLAIWTIVQSILWFVIYFQVVASVMWMLIEGAYLWYLYMACGWGKLPMKFLDGYVNNYVFWILAGTMG------------------TIKATLLLVPLLGVSNIPLFYMLGSAILQHSQQGIFIAVLYCF
Ppa_PPA19689            : ----------------------------------------------------------------------------------------------------WILAGTMGFALIMNLIFLLAIVVILVTIKATLLLVPLLGVSNIPLFYMLGSAILQHSQGIFIAVLYCFL
Min_MiV1ctg289          : KVSLSLSVVSVCVLLAALVLFSAFDKNIQNLAAAFVLRFAILALWTFLNTSLLFGLIYFQVASVMWMLIEGVYLYFVYLFTGWVLPFLVVASWATIHERQWILAGTMG------------------TVKATMLLVPLLGVSNVPLFYMLGSAILQHS---QISQLYCFL
Min_MIV1ctg690          : ------------------------RLLHLHLMIAILAMVLLRLVLYIDLILMFFLLEYFKSVAFWWMFLEQGLFLWPYLLTGYGVPFIHTLGWLIILFIKWILDGPRRRLRVFRNLLHLHLMIAIL-------------------------------------------
Bma_Bm1_40745A          : NVSLTLSIVSVALLVAS-----------------------------------------------VMWMLIEGGYLNYAFLFCGWGVPFVVVVSWTIIHQRRWILSATMGLALILNLLFLLGIVLILVTVKSTLLLVPLLGVSNIPLFYMLGSAILQHSQGIFIAVLYCFL
Bma_Bm115090            : KLEFVGLGLSLISLLISIAIFSYFRLLHLQLMIAILMVVIIRLILYIDLITMFFMLEYFKTVAFCWMFLEGFYLLRRYLITGYDLTGFPLVQTFLWLAVIWLLDGPRMFELVINLFFICNVIRVLW-------------------------------------------
Tsp_EFV57875            : VITLWLSVISAILMIASLATFIKLN--------------------------------------VLWMFLEGAYLFLIYIAVGWGLPVLFVLGWVLALELQWLLIGPMSVALGLNFLFLLVIVVVLVTIKATLLLVPLLGLTNLLLFY----------KGIFVGIFYCFG
Tsp_EFV61832            : NLEFVGLALSFVGLITAIIIFSVFRQVHLQLMLAILLTVVIRLVLYVDQIAFYVALEYGKTVAFAWMFIEGFYLMLKYIIAGWGIPLIHVSVWLIVVIVKWIMDGVRLSELLINFIFLLNIIRVLWSVKAALMLIPLLGTPNILQTITYVASFFYMFQGFMVAVLYCFT

Dme_PDFR                : TLEIVGLCLSLFALIVSLLIFCTFRKIHKNLFVAMVLQVIIRLTLYLDQFASYVLLEYARTAMFMWMFIEGLYLLKFFSRLGWCVPILMTTVWARCTVMYWILEGPRLAVILLNFCFLVNIIRVLVAVRAAIVLLPLLGITNLLHQLSYGTHFLTSFQGFFIALIYCFL
Aae_AAEL009024          : TLEIVGFSLSLIALIISLVIFCRFRRIHKNLFVAMVIQVIIRLTLYIDQAASYILLEYARTCMFMWMFIEGLYLHTLYAMVGWGGPVALTAIWAVTTAQYWILEGPRLAVVLLNFIFLLNIIRVLIAVRAAVVLVPLLGITNLLNMTSYVTHFLTSFQGFFIALLYCFL
Aga_AGAP003654          : TLETVGFSLSLIALIVSLVIFCKFRRIHKNLFVAMVIQVVIRLTVYIDQAASYVLLEYARTCMFMWMFIEGLYLHKLYAIVGWGGPLILTVIWAIITAQYWILEGPRLAAVVLNFIFLLNIIRVLIAVRAAVVLVPLLGITNILNMTSYVTHFLSSFQGFFIALLYCFL
Ada_ADAR2072_2          : --------------------------IHKNLFVAMVIQVVIRLTLYIDQAASYVLLEYARTCMFMWMFIEGLYLHTVYAAVGWGGPVVLTVIWAITTAQNLISPSLPGVCLQLNFIFLLNIIRVLIAVRAAVVLVPLLGITNILNMTSYVTHFLTSFQGFFIALLYCFL
Cqu_CPIJ009749          : TLEIVGFSLSLAALIISLVIFCRFRRIHKNLFVAMVIQVIIRLTLYIDQVASYILLEYARTSMFMWMFIEGLYLHALYAFVGWGGPVALAAIWAITTAHYWILEGPRLAVVLLNFIFLLNIIRVLIAVRAAVVLVPLLGITNILNMTSYVTHFLTSFQGFFIALLYCFL
Ame_GB14562             : TLEFVGLSISLVALFASLAIFCRFRRIHKNLFVAMVVQVLIRLTIYIDIEASYALLEYAKTAMFMWMFIEGLFLYRMYRFIGWGCPVMMTLIWATITAFYWILEGPRFAVILLNFLFLLNIVRVLVAVRAAVVLLPLLGITNVLFMISYSTHFLQSFQGLFIATLYCFL
Nvi_NV24834             : YLEFVGLSISLTALLISLSIFCRFRRIHKNLFVAMVIQVVIRLTLYVDQAASYVLLEYARTAMFMWMFIEGLFLYRTYRLVGWGYPIAMTLTWAIVTAFYWILEGPRMAVILVNFLFLLNIIRVLVAVRAALVLLPLLGITNLISMASYTTHFLTSFQGLFIATLYCFL
Ace_ACEP15240           : --------------------------------------------------------------MFMWMFIEGLFLYRMYRFIGWGCPVLMTSAWAIVMAFYWILEGPRFAVILLNFLFLLNIIRVLVAVRAAVVLLPLLGITNMLFMISYSTHFLRSFQGLFIAMLYCFL
Tca_TC013682            : TIEMVGLSFSLISLIVSLIIFFQYRKIHKNLFIATLLQVVFRLIKYVDQEACITLLEYSKTAMFTWMFIEGLYLIKIYFYVGWTAPAVITAVWVWTMKMVWILEGPRSTIIVVNLLFLINIIRVLIAVRAAIFLLPLMGIANILFWMSYTSYFLN--------------
Bmo_BNGRB2              : YLEIFGFTLSFIALSISLYIFIHFRRIHKHLFGAMLVQVLIRLTVYIDQAGSYVLLEYATSAMFLWMFMEGLYLYQWYCVWAWGAPVVITSIWTILTALKWIVQGPRLIVILINFVFLLNILRVLIAVRAALVLLPLLGITNIMNMFSYLTHFLRSFQGFFIALIYCFL
Dpl_KGM08731            : --------------------------------------------------TCYVIMECTCSAMFAWMLIEGIYIFKIYCWFAWGVTIFFTTVWAFSTKPNWIMQAPRFVVIMINMVTLVLILKRLYSVKVVTILTPLLGIVNILNMILYVSHFLRSFQGCFLSLFYCLF
Hme_HMEL012068          : YIEITGYIISFIAIIVALYIFKKFRRIHKHLFYSLLFETV----------VLYVMAEYSVAAVYSWIFIEGLHLFKTYCICGWTVPLLLTTIWTAVVWISWILQGPRVTVILCNAVILVRVMKMVISVKAALFLLPILGLSNVLSIFCYTRQFIKSFQGFLLTLIYCVD
Api_ACYPI46431          : VIEVVGCLVSIISLIISLCIFIRHKKIHMNLFVALSLQMFIKLILNIDKLGSQVILEWSKTASFMWMYNEGVYLNFRMYLFGWGLPALMTSVWLLVTMVYWILEGPRMIILVVNLIILIIVLRELLVSKAAIALVPLLGITNFILVISYSAHILHSFQGLMVSILYCFM
Rpr_RPRC009680          : TLEIYGFSISLAALFISLYIFSHFRKIHKNLFAAMVAQAVIRLTLYVDQAASYVLLEYARTAMFMWMFIEGLYLYKLYTSLGWGAPVIMTSAWAVTLAVQWILEGPRFAVVILNFLFLLNIIRVLVAVRAAVVLLPLLGITNLANMLSYATHFLTSFQGFFVAALYCFL
Phu_PHUM127410          : TLEIVGFSVSLIALLFSLAIFFRFRRIHKNLFIAMEIQVVVRLSLYIDQVISYILLEYARTAMFMWMFVEGLYLYTMYSIIGWGSPIIITSAWILVMITYWILEGPRLTVVVLNFLFLLNIIRVLVAVRATAVLLPLLGITNIISMSSYGTHFLTSFQGFFIAFLYCFL
Dpu_DappuP309887        : VLELTGLTVSLISLMISLFIFTYFRRIHKNLFAAIGIQVIIRMTLYLDQAWFYIVLEYARTTMFVWMFIEGLFLHKLYLALGWGIPIILTAIWAAFTATHWILEGPRLTIIFINLLYLLNILRVLVSVKAAMVLLPLLGITNALVMISYSSHFLTSFQGFFVALLYCFL
Isc_ISCW017309          : YILLLFSLLSLIFLFATMFIFCYFRRVHQNLVLALMVHAVMLVVLSLPVVSILSLKMYAAMASINWMFVEGLLLFKLYHAIGWGLPLTFILSWAYLMEQTWLLIGPRLVALLVNFVFLVNIIRILVAIKATVLLFPLLGITHLLFCIMIINAILQSSQGIFVSVLYCFM
Isc_ISCW017314          : --------------------------------------------------SILSLKMYAAMASINWMFVEGLLLFKLYHAIGWGLPLVFIVAWAYLM---WLLIGPRLVALLVNFVFLVNIIRILVAIKATVLLFPLLGITHLLFCI----------------------
Tur_tetur04g08940       : NWTNYTQCLSLLSSFSFPLEFSRLQIIHRNLVFALIIHCVSLLIISSSIVLVLSLKMYSALASINWMFIEGLLLFKLYYAIGWGIPLFIILTWAYIVNKTWIITAPMILALLINSTFLVNIIRILVALKATILLFPLLGITHLLFCFMITNAILQSSQGIFVSFFYCFG
Tur_tetur317g00010      : ----------------SSELECPRLRVHRNLVFALIIHCVSLLIISSSIVLVLSLKMYSALASINWMFIEGLLLFKLYYAIGWGIPLFIILTWAYIVNKTWIITAPMILALLINSTFLVNIIRILVALKATILLFPLLGITHLLFCFMITNAILQSSQ-----------

Spu_XP_003729511.1      : ILAIVGNSISWISLLVALCIFMHFRKIHKHLFVSLLIRLTIEVIFAVDRHIFEVLREYGRFCAFAWMFIEGMYLFLLYYLIGWVFPIPFVSAWAIAMEMTGLIEVPRNVLLAINAIFLINIVRILVAVKAAIVLLPLLGIVNLLWVLLFVFRFFAEFQGFFVALLY---
Lva*                    : ILAIVGNSISWISLLVALCIFLHFRKIHKHLFVSLLIRLTIEVIFAIDRHIFEVLREYGRFCAFAWMFIEGMYLFLLYYLIGWVFPIPFVSAWAIAMEMTGLIEIPR---------------RVLVAVKAAIVLLPLLGIVNLLWVLLFIFRFFAEFQGFFVALLY---
Sfa*                    : ILAIVGNSISWIS---IIIIFDFSHKIHKHLFVSLLIRLTIIEYAVHVFFIFEVFREYGRFCAFAWMFIEGMYLFLLYYLIGWVFPIPFVSAWAIAMEMT---------------------------------------------------------------------
Afr*                    : ILAIVGNSISWISLLVALCIFLHFRKIHKHLFVSLLIRLTIEVIFAVDRHIFEVFREYGRFCAFAWMFIEGMYLFLLYYLIGWVLPIPFVSAWAIAMEMTGLIEVPR-------------------AVKAAIVLLPLLGIVNLLWVLLFVFRFFAEFQGFFVALLYCFL
Pmi*                    : -------------------------KIHKHLFAAYIARLTIEIILGVNRFTFETIREYGRLCAFFWMFIEGLYLFLLYYFIGWRK-----------------------------------------AAKGAVLLIPLLGVANLAWFINYLFLFLDSYQGFFLCVMYCFL
Sko_115725_115726+      : ILEIVGYSISLACLLVAFFIFCYFRTIHKQLFLSLLIRTVLDIILYVDNTTFEYFRQYTRFCVFSWMFVEGLYLFKLYYFIGWILPLFPVTAWAITMKFTWIVEIPRNLILLINLGFLINIIRILVAVKAAILLAPLLGVTNLLFLPFYGVMIVTPFQGVIVSLLHCFV
Bflo_110555             : CIYFVGSSISLILLAATFFIFCYFRKIHKHLVLSLIFRSIVLIVLLQPYCRMLVLSQYFGMTNVFWMLIEGLFLYMLFYIIGWVVPVCFVAAWTVVMCW-WIVSGPIVLALLVNLYVLLHILAVLVTLKALFVLLPLLGLTNVLFFVPIFQVFNAVIQAEGIFVSMIYC
Bflo_66998              : CIYVVGSSLSLLTLCVTLFIFCYFRRIHKHFVLSLILRAIILIVLLQPFCRLVVLLQYFSLTNIFWMLIEGLFLYVLFYVIGWGAPVVFVAVWTVVMCW-WIISGPIVGALLVNLYFLFHILAVLVTLRALFVLLPLLGLTNLLFFVPIFQIFNALLQSQVKSVIRDRM
```

* Retrieved from concatenation of short 454 or EST contigs
+ from Contig115725 and Contig115726

```
                          TM1                      TM2                      TM3                      TM4                      TM5                      TM6                      TM7
```

Cluster A receptors

```
Cel_Seb-2              : LAGLLTYSASVIFLIPAVFLLTLLRILHRHLLISCLLYGAFYLITVSLFVLFSIQLRYLRLTNFTWMLAEAVYLLRSYKVICWGVPGVITVVYIFVRSLNIITPSLLAMGVNL-----LLLGLIVYAVRGALMLIPVFGVQQLLTIYQVTDQSLNGLQGMFVSFIVCYT
Hco_Hc_scaffold1076    : LAGIIAFSISVVCLIPAIFILWFFRIVHRHLLTSFVLSGLFYLFNCFFFVLFLVQLRFLRLATFSWMLAEGVHLLTIYKLLCWGVPAVIAVVYGVLRESLIIIP----------------------AVRAAVILIPVFGLQFLFTIYAVILIPVFGLQFLFTIYRLSIE
Ppa_PPA02324           : FAGVFSYAISVIAIIPAVVILSVFRVIHKHLLISFLLLGIFYLFTSFMFILFLTQLRFLRLSTFSWMLAEGVYLLIVYKIVCWGFPLLISIVYGVLRLQLVMFPSLFCITLNL-----ILVTVILYAVRAALMLVPVFGLHFLFTIYQIMNLIMDGLQGFAVSIILCYA
Tsp_EFV58944           : YYIYWIGYTVSLVVLLTSLAIFIKF------------------------------------------------------------------------------------------------------------------------------------------------

Tca_TC001222           : ---IIVLSVAAVLSAPAVAIFYSFRALHRNLILVIIIKNLLVVITKESRFVYCAFFVLSCCPALIWALLRGLHT-----------------------------DAFRLTILTVNFVLLVDIIRVLKTLRVTLFLVPLFGVHIVITVYYFATYLIEGLQGVLVAFLFCY-
Tca_TC001223           : ---VIVLAVATVLSFPAVAIFFSFR------------------------------------------------------------------------------------------------------------------------------------------------
Bmo_BNGRB3             : RFHIAMLSISVASCLPAVFIFFFYKALHRNLLIAIILRNTFVIISRNEIYFLAIGERVAGNAVFVCMLVEGIYLIKSLYAIGAVIAVAPVIAWVAVMAVHWILDVPRVAILLVNTVLFGDILRVLLTTKATLFLMPLFGTQFLLTAFYYVSYTVEGLQGFVVAILYCYI
Dpl_KGM06692           : KFYVAMLGVSVVTCLPAVFIFFFYKALHRNLLIAIIIRNIFVIISRSAIYVVSIFERAASNAVFVCMLVEGIYLIKSLYAIGWPLPSVPSGIYVVVKSPRWSLDGPRVLVLLVNTLLFADVLRVLLTTKATLFLIPIFGTQFLFTAIYFVAYSIEGLQGFIVAMLYCYV
Hme_HMEL008977         : RFHIAMLALSTVACVPAVCIFLFYKALHRNLMIAIIVRNVLVMISRNEIYVLAVAERTAANAVFLCMLVEGIYLFSARFYLLWVLPLFPTGIYAIIRSIWWALDAPRLFILVVNTVLFIDVLRVLLTAKATLFLMPIFGTQFIFTAIYFIAYVMEGLQGFVVAMLYCYV
Phu_PHUM15970          : YVTIGSYALSVIALVPALIIFQKFRILHKNLFASLLFHCSTQIAFNSEFIALLVATKYFRLTNYFWMFCEGFYLYTMYSIIGWGVPIIPVAIFIAFRKAIWALHLPCLLSLLVLLIFLVNIIRILVSVKATLVLVPLFGIHFLFTLYAYIRSTFDGLQGFLVAVIFCYV
Dpu_DappuP328650       : HVHLYAYAVSVATLLPAIFIFYSYKRLHKNMFISILLNSILVIVFKVFMILMLITVKYLRLTNYAWMFCEGFYLFSVFYVIGWVLPLFPTGIYAIIRSIWWVLNTPCLFSLVVNLIFLVNIIRILVAVRATLVLVPLFGLHFVVTIYHYANALLDGLQGFLVAIIFCYG
Isc_ISCW003092         : HYSIGLLALSVLSLIPALVVFNWYKSIHKHLCFSVMMCGTFYIIDHAVFTLLTALNRYFMVSQYSWMLCEGCYLLKVYYGVGWGAPVVLVIIYSVLRATKYVFTGPILAALAVNIFFMTHVVYILVVLRAVLILIPIFGCHFILTTFQYAEWTIVGLQGLFVSLIFCYF
Isc_ISCW018841         : ---IILHSVSALLLLPAIIIFSFYAQMHKHLCTSLLLYGIASIAIDYVLI------------------------VLLYYSVGWGFPAVFLATYAVFRAK----------------------------------------------------------------------
Isc_ISCW017538         : --------SSPKVLVPAIVIFSVYK-------------------------VLYTLSRYFRLCQYAWMFCEGFYLLLIFYVVGWGCPAIFVTISAVLRAMR-----------------------------AVLVLFPLFGMHFLMTVY----------------------
Tur_tetur18g02640      : RYLIVTHLTSVTFIIPALYIFLYYRKIHVHLLISLLSYSILMSLLTLDMICLSVLVKYFRSTTYFWMFNEAFYLLKSLIALAYGVPLLTNTSYIIVRFISWIINGPNLSVLVINFIFLISVLREICSLRAASLLLPLYGLHYLFIVYHYASITLDGLQGSIVSILYCFL
```

Cluster B receptors*

```
Tsp_EFV57580           : LIGVIGYCASLVSLSLASAVFLHFKAFMCLLQYSLFRNGKTTLFDGDLTISIITMWNYSLIASYFWQLMEGMYLVILYVLLGWGLPMIIMVPWIVVRLKDLRG--PIMLTIVINFILCMNICR----------------------------------------------

Ame_GB10993            : IISKIGYTVSFFTLVIAFFILTVIKILHMHLFASFMFRAFMALMKDIVFVMFTSFWQYFILANYFWILMEGLYLIAVYICLGWGLPALFVLCWIVARLFIFLFRIPTMLSILINFVLFVNIVRVLLWAKSTLVLVPLFGVHYTLFLGLFCDQLFASFQGFFVAVLYCFL
Ame_GB30248            : VATTMATKVCLWYSNPENIRESLCERVNLLGLRSRDYKNFESLLNKWLPVFASFIMRAFMTLVKDWTFMEGLYLISLYVLMGWGLPVFVVVPWIIIRATILLIRIPIMISNLFNFLLFLNIVRALFWAKSTLVLVPLFGAHYTLFLGLFFDQLFVSFQGSFVALLYCLL
Nvi_NV24662            : ILDQIGYGISILSLVIALSIFAIFSKVHINLFTAMLLQSLMSLTQNFFYGTLMTLSHYFELTIFTWLLIEGWYFIIPHILAGWGMPLTVMIPWVLVHIYSNIVAIPILLIVLVNLCFFILNVRIIFWIKSSLMLVPLFGTYDILYGILLIVRLICSFQGCIVAALYCFS
Nvi_NV24008            : MVVRLGYAVSTFALSIALSIFTLRPKIHTHFFVSLALQAITWLTQGMIYGALMTLGNYFELCSFSWILMESWYFVKPLIICGWGLPVIVMIPYLITYSLANIVAVPILLMVLINCSFFVLNVKVLFWFRSTLMLVPLFGAHDIFFCMYNAVRVVCAFQGLLVSILYCFI
Nvi_NV11142            : VTSQLIYLLSFLVLAVALVLFLLFKRLHMHMLSATIMQVVMWLMHSVLFDVLAGLERYFETTSLFWLLMEGWFLIKRYVIIGWGVPIIVTSPWMVYHVYSYLIAVSAFLTFVINLYFFVEISKVLYWVRSTFMLVFLFGAQNALYVAASFQSNKPRLQGIFVAFIYCFL
Nvi_NV16892            : CISKAGYAVSLSALVVAFCILASIKILHMHLFASFMVRAFTFLLKNLLFVAFTSIWQYCILANYSWILMEGLYLIAVYVVLGWGLPAVFVTTWVILRAVFQIIRLPTTFSVVINFILFVNIVRVLMWARSTLVLVPLFGVHYSIFLGLFCDQLFASFQGFFVAVLYCFL
Ace_ACEP10203          : -------------------------RLHMHLFASFIMRAFMALLKDWSFIIITSLWQYFIVANYSWTLMEGLYLIFLYIVLGWGLPVLVVVPWIIFRATILIIRIPMIICILFNFLLFLNIVRVLLWAKSTLVLLPLFGIHYTIYLGLFCDQLFASFQGAFVAFLYCLL
Tca_TC008110           : NITQCGYILSTVSLIISLFVFIRIKKLHIHLFASFVMRALMSLIKDGLFIAIISLWNYFIISNYMFLLMEGAYLVVIYYSLGWGIPLLFIIPWIVLKAGNMLIDVPIGLTVVINFILFTIIVRILFLIRAILILVPLFGIPYTISFVLFFDQTFTAFQGLFASLVYCLL
Tca_TC010267           : VISETGYIVSFATLIIAFAIMLFIKILHMHLFASFILRALTFIVIKSTFVLLTSLWQYFITANYSWILMEGLYLIKWYVVMGWGLPLIIVGFWVAARLLVLIIGIPTMVSILINLLLFMRISMVLYWVKSTLVLVPLFGVHYALFLALFCDLLFGSFQGFFVAILYCFL
Rpr_RPRC011086         : -----------------------------------------------------------------------------------------------------------------------------MWAKSTLLLVPLFGVHYALLIGLFCEQLFASFQ-----------
Phu_PHUM23900          : GISHIGYGISLISLVMASLILLSFRTLHLHLFVSFIMRAFVTFLKEFLFESLTTIWQYFIMANYSWILMEGLYMIFMYIILGWGLPIIFVISWALVRINLLIIRIPIIISVLVNFGLFLNIIRLLLLAKSTIVLAPLFGVHYTIFLGLFCDQLFASFQGFFVALFFCFL
Dpu_DappuP41513        : RISQVGYGVSLGTLLIALCILASFKTLHMHLFISFIMRAFMALLRDNLFVLVTSLWQYFIMANYSWILMEGLYLITIYIILGWGLPFIFVSSWVVMRIIFLLIRGPITASILVNLIFFLHIAWVLLWAKSTLVLVPLFGVHYTVYCILVSDQLFASFQGFLVALLYCLM
Isc_ISCW014021         : -ISQVGYGVSLGTLLIALCILASFKTLHMHLFISFIMRAFMALLRDNLFVVFTSFWHYVLMANYCWILMEGLYLILRYVALGWG-------------------------------------------------------------------------------------
Tur_tetur10g00830      : LISKIGYSVSLLTLIIAFLILFCNKHLHLQLFLSFISRSLLTHVK-----LIIVVWQYSLLANYNWLLMEGLYLILKYIILGWSLPVLFIIPWIIARSLYWIIRGPITISIILNLIFFINITRVLFWFKSTLVLVPLFGVHYALLLILYVDQLFTSFQGFFVALLYCFL
```

*no TM domains were identified for Ppa_PPA19772
