# Supplementary figures and images for: Nematode and Arthropod Genomes Provide New Insights into the Evolution of Class 2 B1 GPCRs
Source: PLoS One. 2014 Mar 20;9(3):e92220. doi: 10.1371/journal.pone.0092220 (PMC3961327; doi:10.1371/journal.pone.0092220)

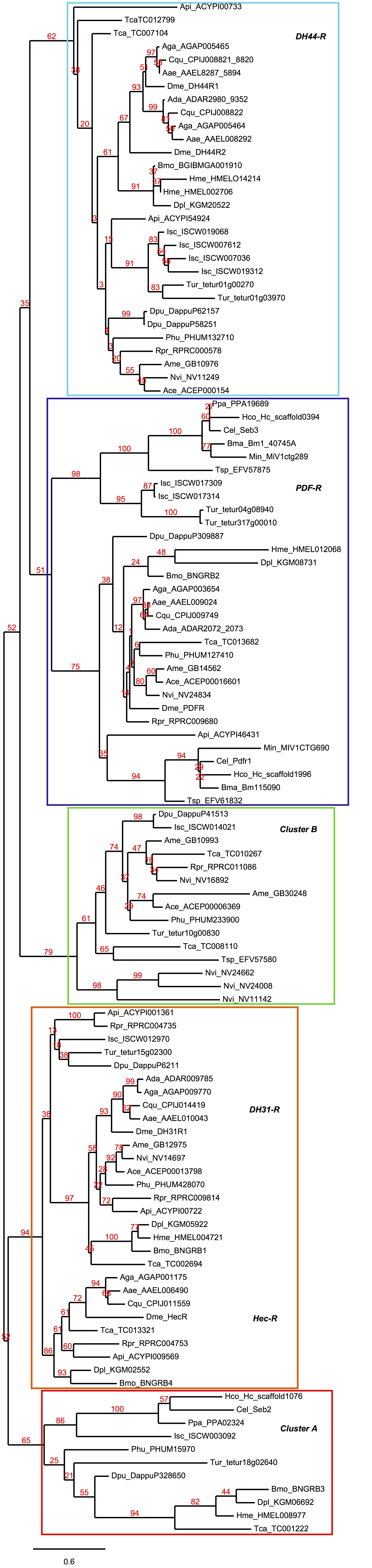

Supplement: Figure S2 — Maximum likelihood phylogenetic tree of the nematode and arthropod Class 2 B1 receptors. Reliability of internal branches is assessed using the bootstrapping method (100 bootstrap replicates). Analysis is based on the amino acid sequence alignment of the Class 2 B1 receptors TM regions and included 116 nematode and arthropod sequences. (TIFF) [file pone.0092220.s002.tiff]

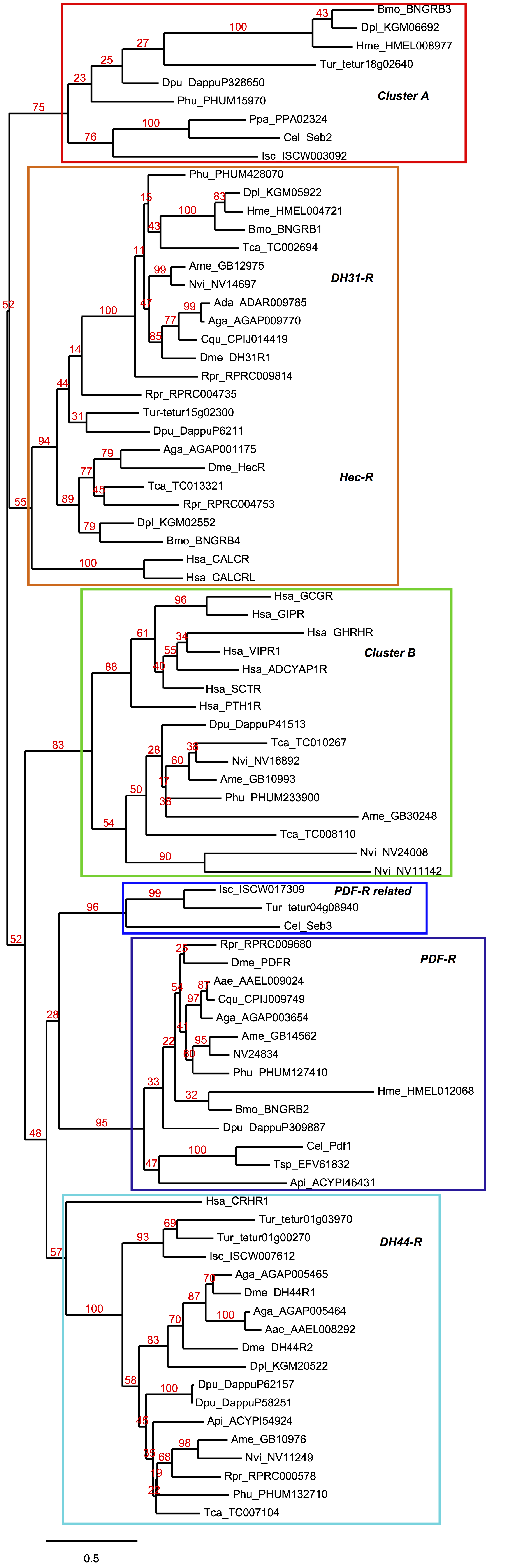

Supplement: Figure S3 — Maximum likelihood phylogenetic tree of the nematode, arthropod and human Class 2 B1 receptors. Reliability of internal branches is assessed using the bootstrapping method (100 bootstrap replicates). Analysis is based on the amino acid sequence alignment of the human Class 2 B1 receptors with the nematode and arthropod receptor genes possessing the seven TM regions. (TIFF) [file pone.0092220.s003.tiff]

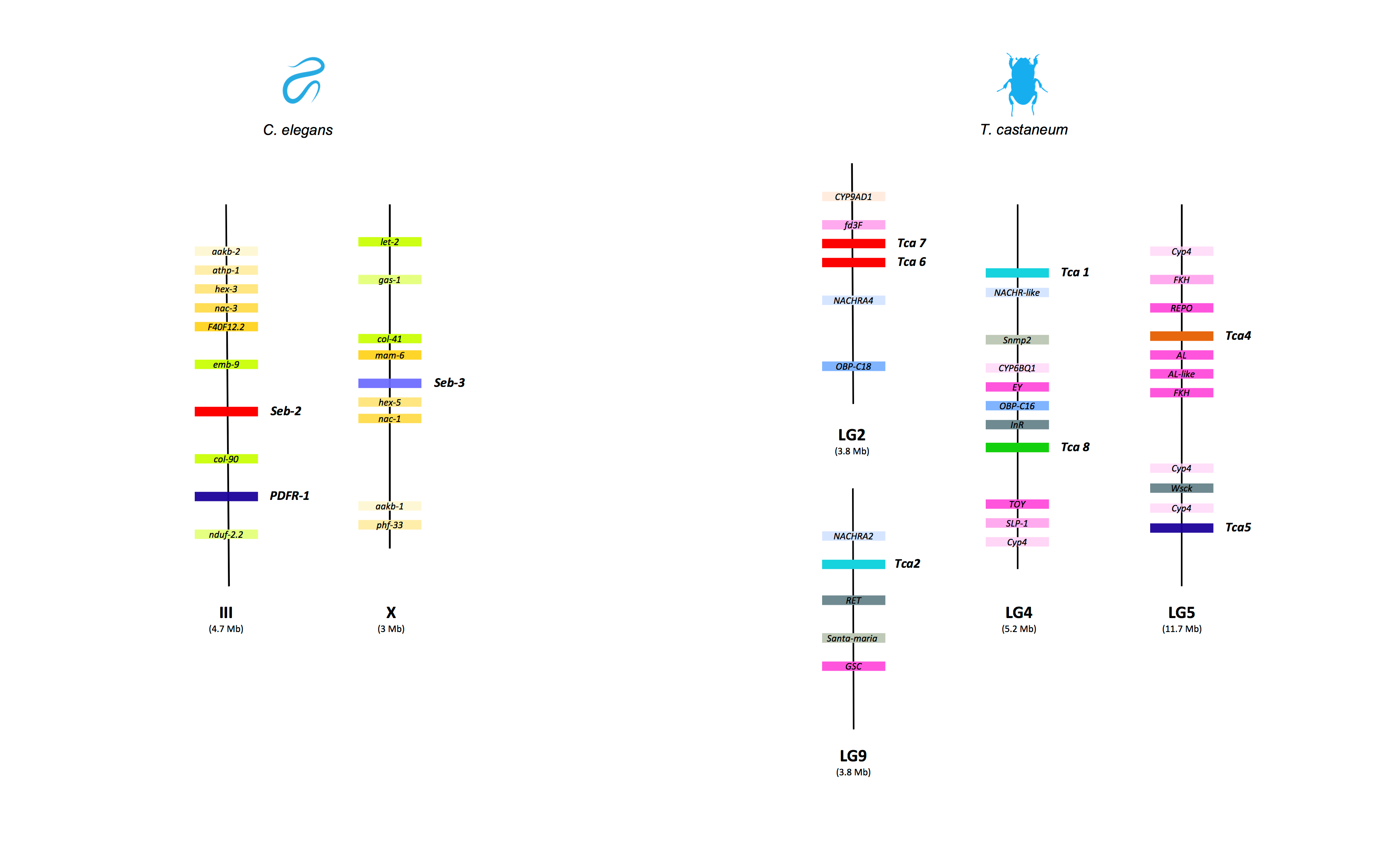

Supplement: Figure S4 — Gene environment comparisons of Class 2 B1 receptors in C. elegans (A) and T. castaneum (B) chromosomes. Gene symbols were obtained from ENSEMBL annotation and when not available for T. castaneum the homologue designation in D. melanogaster was used. Solid horizontal lines represent chromosome fragments and genes are represented with blocks. To facilitate visualization genes are colored according to which family they belong. The position of Class 2 B1 receptor genes within the C. elegans and T. castaneum chromosomes are annotated in color according to the phylogenetic analysis obtained from Figure 1. Lengths of the extracted chromosome regions that contain the Class 2 B1 GPCR genes are indicated (Mb). Only genes common between the species analyzed are represented. (TIFF) [file pone.0092220.s004.tiff]

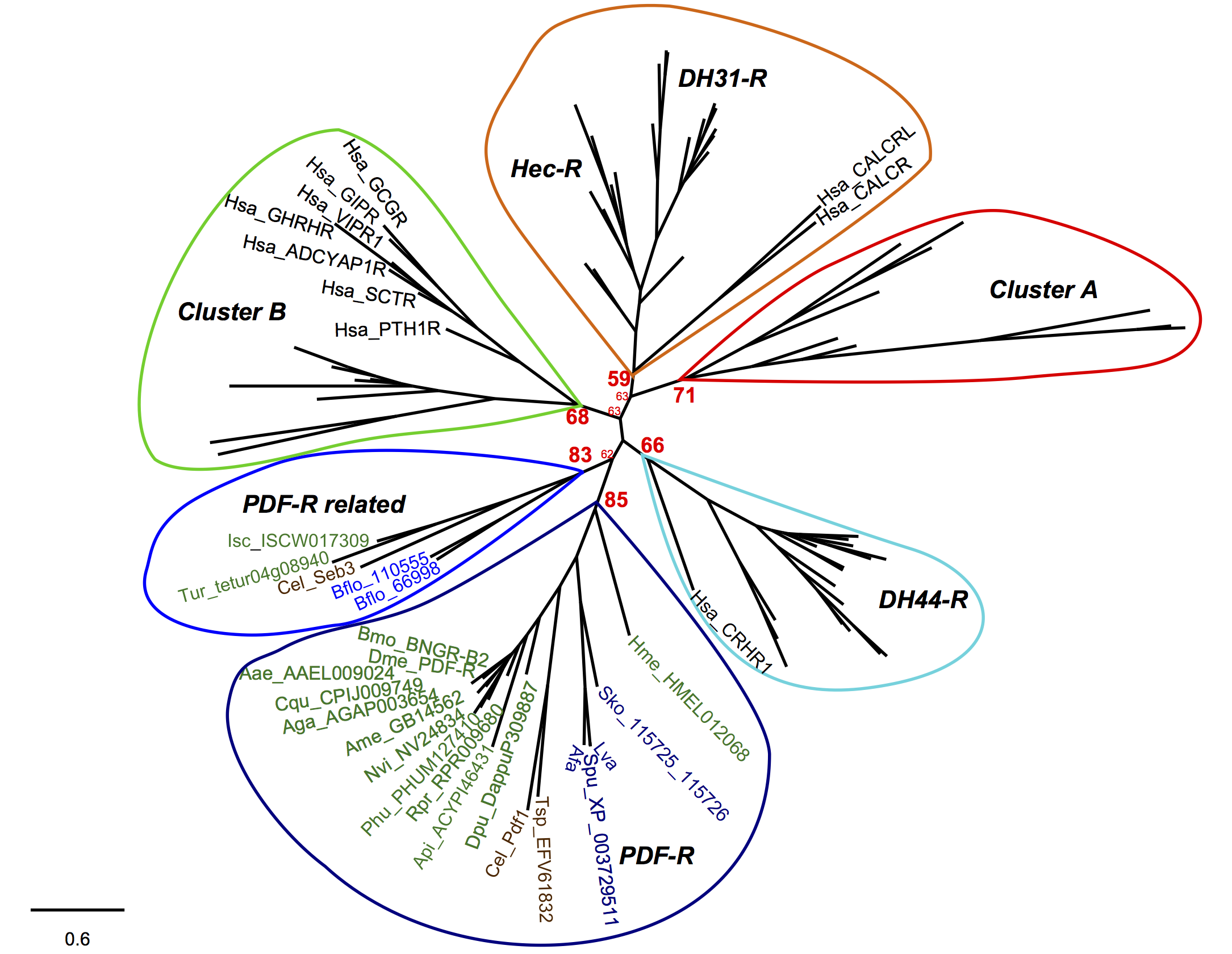

Supplement: Figure S5 — Maximum likelihood phylogenetic tree of the nematode, arthropod and human Class 2 B1 receptors with the putative early deuterostome PDF-R-like receptors. Reliability of internal branches is assessed using the bootstrapping method (100 bootstrap replicates). Analysis is based on the amino acid sequence alignment of the nematode and arthropod Class 2 B1 receptors with the seven TM regions. (TIFF) [file pone.0092220.s005.tiff]
