# Supplementary material for: Nematode and Arthropod Genomes Provide New Insights into the Evolution of Class 2 B1 GPCRs
Source: PLoS One. 2014 Mar 20;9(3):e92220. doi: 10.1371/journal.pone.0092220 (PMC3961327; doi:10.1371/journal.pone.0092220)
Supplement: Table S1 — Accession numbers of Class 2 B1 receptor genes extracted from the nematode and arthropod genomes analyzed. Nematodes are shaded. Genes annotated with an asterisk (*) indicate sequences that were not included in the invertebrate (blue, Figure 1) and invertebrate-human (red, Figure 2) phylogenetic tree analysis. (PDF) [file pone.0092220.s006.pdf]

Table S1

	**DH44-R**	**DH31-R**	**Hec-R**	**PDF-R**	**Cluster A**	**Cluster B**
C. elegans	*n.i.*	*n.i.*	*n.i.*	Seb-3<br>Pdf	Seb-2	*n.i.*
H. contortus	*n.i.*	*n.i.*	*n.i.*	Hc_scaffold0394*<br>Hc_scaffold1996*	Hc_scaffold1076*	*n.i.*
P. pacificus	*n.i.*	*n.i.*	*n.i.*	PPA19689*	PPA02324	PPA19772**
M. incógnita	*n.i.*	*n.i.*	*n.i.*	MiV1ctg289*<br>MiV1ctg690*	*n.i.*	*n.i.*
B. malayi	*n.i.*	*n.i.*	*n.i.*	Bm2293*<br>Bm2168*	*n.i.*	*n.i.*
T. spirallis	*n.i.*	*n.i.*	*n.i.*	EFV57875*<br>EFV61832	EFV58944**	EFV57580**
D. melanogaster	CG8422<br>CG12370	CG32843	CG4395	CG13758	*n.i.*	*n.i.*
A. aegypti	AAEL008292<br>AAEL8287_5894[a]*	AAEL010043*	AAEL006490	AAEL009024	*n.i.*	*n.i.*
A. gambiae	AGAP005464<br>AGAP005465	AGAP009770	AGAP001175	AGAP003654	*n.i.*	*n.i.*
A. darlingi	ADAR2980_9352 [a]*	ADAR009785	*n.i.*	ADAR002072_2073**	*n.i.*	*n.i.*
C. quinquefasciatus	CPIJ008821_8820 [a]*<br>CPIJ008822*	CPIJ014419	CPIJ011559	CPIJ009749	*n.i.*	*n.i.*
A. mellifera	GB10976	GB12975	*n.i.*	GB14562	*n.i.*	GB10993<br>GB30248
N. vitripennis	NV11249	NV14697	*n.i.*	NV24834	*n.i.*	NV24662<br>NV24008<br>NV11142<br>NV16892
A. cephalotes	ACEP00015444*	ACEP00013798*	*n.i.*	ACEP00016601*	*n.i.*	ACEP00006369*<br>ACEP00010987**
T. castaneum	TC007104<br>TC012799*	TC002694	TC013321	TC013682*	TC001222*<br>TC001223**	TC008110<br>TC010267
B. mori	BGIBMGA001910*	BGIBMGA009927	NP_001127735	BGIBMGA012242	BGIBMGA012453	*n.i.*
D. plexippus	KGM20522	KGM05922	KGM02552	KGM08731	KGM06692	*n.i.*
H. melpomene	HMEL014214*<br>HMEL002706*	HMEL004721	*n.i.*	HMEL012068*	HMEL008977	*n.i.*
A. pisum	ACYPI00733 *<br>ACYPI54924	ACYPI007222*<br>ACYPI001361*	ACYPI009569	ACYPI46431	*n.i.*	*n.i.*
R. prolixus	RPRC000578	RPRC009814	RPRC004753	RPRC009680	*n.i.*	RPRC011086*

		RPRC004735				
P. humanus	PHUM132710	PHUM428070	*n.i.*	PHUM127410	PHUM15970	PHUM233900
D. pulex	DappuP62157<br>DappuP58251	DappuP62111	*n.i.*	DappuP309887	DappuP328650	DappuP41513
I. scapularis	ISCW019312*<br>ISCW007036*<br>ISCW007612<br>ISCW019068*	ISCW012970*	*n.i.*	ISCW017309<br>ISCW017314*	ISCW003092<br>ISCW018841**<br>ISCW017538**	ISCW014021*
T. urticae	tetur01g00270<br>tetur01g03970	tetur15g02300	*n.i.*	tetur04g08940<br>tetur317g00010*	tetur18g02640	tetur10g00830*

n.i.; not identified ; [a] Sequence obtained from the combination of two predicted genes (AAEL8287_5894 – AAEL008287 and AAEL005894; ADAR2980_9352 - ADAR002980 and ADAR009352; ADAR002072_2073 – ADAR002072 and ADAR002073; CPIJ008821_8820 – CPIJ008821 and CPIJ008820).
