# Supplementary material for: Nematode and Arthropod Genomes Provide New Insights into the Evolution of Class 2 B1 GPCRs
Source: PLoS One. 2014 Mar 20;9(3):e92220. doi: 10.1371/journal.pone.0092220 (PMC3961327; doi:10.1371/journal.pone.0092220)
Supplement: Table S2 — List of the accession numbers and adopted acronyms of the nematode and arthropod Class 2 B1 GPCR genes. The symbols of the genes previously identified (C. elegans, D. melanogaster and B. mori) were maintained. (PDF) [file pone.0092220.s007.pdf]

Table S2

	Genes	Acronyms
Nematodes		
C. elegans	C18B12.2	Seb-3
	C13B9.4	Pdf
	ZK643.3	Seb-2
H. contortus	Hc_scaffold0394	Hco1
	Hc_scaffold1996	Hco2
	Hc_scaffold1076	Hco3
P. pacificus	PPA19689	Ppa1
	PPA02324	Ppa2
	PPA19772	Ppa3
M. incógnita	MiV1ctg289	Min1
	MiV1ctg690	Min2
B. malayi	Bm2293	Bma1
	Bm2168	Bmal2
T. spiralis	EFV57875	Tsp1
	EFV61832	Tsp2
	EFV58944	Tsp3
	EFV57580	Tsp4
Arthropods		
D. melanogaster	CG8422	DH44-R1
	CG12370	DH44-R2
	CG32843	DH31-R
	CG4395	Hec-R
	CG13758	PDF-R
A. aegypti	AAEL008292	Aae1
	AAEL8287_5894	Aae2
	AAEL010043	Aae3
	AAEL006490	Aae4
	AAEL009024	Aae5
A. gambiae	AGAP005464	Aga1
	AGAP005465	Aga2
	AGAP009770	Aga3
	AGAP001175	Aga4
	AGAP003654	Aga5
A. darlingi	ADAR2980_9352	Ada1
	ADAR009785	Ada2
	ADAR002072_2073	Ada3
C. quinquefasciatus	CPIJ008821_8820	Cqu1
	CPIJ008822	Cqu2
	CPIJ014419	Cqu3
	CPIJ011559	Cqu4
	CPIJ009749	Cqu5
A. mellifera	GB10976	Ame1
	GB12975	Ame2
	GB14562	Ame3
	GB10993	Ame4
	GB30248	Ame5
N. vitripennis	NV11249	Nvi1
	NV14697	Nvi2
	NV24834	Nvi3
	NV24662	Nvi4
	NV24008	Nvi5
	NV11142	Nvi6
	NV16892	Nvi7
A. cephalotes	ACEP00015444	Ace1
	ACEP00013798	Ace2
	ACEP00016601	Ace3
	ACEP00006369	Ace4
	ACEP00010987	Ace5
T. castaneum	TC007104	Tca1
	TC012799	Tca2
	TC002694	Tca3
	TC013321	Tca4
	TC013682	Tca5
	TC001222	Tca6
	TC001223	Tca7
	TC008110	Tca8
	TC010267	Tca9
B. mori	BGIBMGA001910	Bmo1
	BGIBMGA009927	BNGR-B1
	NP_001127735	BNGR-B4
	BGIBMGA012242	BNGR-B2
	BGIBMGA012453	BNGR-B3
D. plexippus	KGM20522	Dpl1
	KGM05922	Dpl2
	KGM02552	Dpl3
	KGM08731	Dpl4
	KGM06692	Dpl5
H. melpomene	HMEL014214	Hme1
	HMEL002706	Hme2
	HMEL004721	Hme3
	HMEL012068	Hme4
	HMEL008977	Hme5
A. pisum	ACYPI00733	Api1
	ACYPI54924	Api2
	ACYPI007222	Api3
	ACYPI001361	Api4
	ACYPI009569	Api5
	ACYPI46431	Api6
R. prolixus	RPRC000578	Rpr1
	RPRC009814	Rpr2
	RPRC004735	Rpr3
	RPRC004753	Rpr4
	RPRC009680	Rpr5
	RPRC011086	Rpr6
P. humanus	PHUM132710	Phu1
	PHUM428070	Phu2
	PHUM127410	Phu3
	PHUM15970	Phu4
	PHUM233900	Phu5
D. pulex	DappuP62157	Dpu1
	DappuP58251	Dpu2
	DappuP62111	Dpu3
	DappuP309887	Dpu4
	DappuP328650	Dpu5
	DappuP41513	Dpu6
I. scapularis	ISCW019312	Isc1
	ISCW007036	Isc2
	ISCW007612	Isc3
	ISCW019068	Isc4
	ISCW012970	Isc5
	ISCW017309	Isc6
	ISCW017314	Isc7
	ISCW003092	Isc8
	ISCW018841	Isc9
	ISCW017538	Isc10
	ISCW014021	Isc11
T. urticae	tetur01g00270	Tur1
	tetur01g03970	Tur2
	tetur15g02300	Tur3
	tetur04g08940	Tur4
	tetur317g00010	Tur5
	tetur18g02640	Tur6
	tetur10g00830	Tur7
