# Supplementary material for: Nematode and Arthropod Genomes Provide New Insights into the Evolution of Class 2 B1 GPCRs
Source: PLoS One. 2014 Mar 20;9(3):e92220. doi: 10.1371/journal.pone.0092220 (PMC3961327; doi:10.1371/journal.pone.0092220)
Supplement: Table S3 — Percentage of amino acid sequence similarity of the nematode and arthropod cluster A members. Comparisons were performed using at least 6 TM domains (Figure S1). Nematode sequences are shaded. (PDF) [file pone.0092220.s008.pdf]

Table S3

	Hco3	Ppa2	Tca6	BNGRB3	Dpl5	Phu4	Hme5	Dpu5	Isc8	Tur6
**Seb-2**	64%	78%	43%	42%	43%	52%	43%	52%	57%	58%
**Hco3**		71%	40%	43%	42%	50%	42%	50%	52%	47%
**Ppa2**			45%	48%	47%	51%	50%	55%	60%	59%
**Tca6**				52%	62%	38%	62%	50%	44%	48%
**BNGRB3**					76%	41%	75%	64%	55%	62%
**Dpl5**						45%	92%	57%	53%	64%
**Phu4**							46%	52%	47%	61%
**Hme5**								56%	49%	61%
**Dpu5**									63%	68%
**Isc8**										55%
