# Supplementary material for: Nematode and Arthropod Genomes Provide New Insights into the Evolution of Class 2 B1 GPCRs
Source: PLoS One. 2014 Mar 20;9(3):e92220. doi: 10.1371/journal.pone.0092220 (PMC3961327; doi:10.1371/journal.pone.0092220)
Supplement: Table S4 — Percentage of amino acid sequence similarity of the nematode and arthropod cluster B members. Only arthropod receptors with more than 6 TM identified were used with the exception of Tsp4 (marked with “*” in which 5 TM domains were considered (Figure S1). The nematode representative is shaded. (PDF) [file pone.0092220.s009.pdf]

Table S4

	Ame5	Ame4	Nvi4	Nvi5	Nvi6	Nvi7	Ace4	Tca8	Tca9	Phu5	Dpu6	Tur7
**Tsp4***	35%	41%	39%	38%	35%	42%	36%	44%	41%	47%	44%	44%
**Ame5**		64%	52%	51%	53%	61%	67%	57%	60%	65%	61%	62%
**Ame4**			65%	63%	61%	84%	70%	73%	79%	80%	79%	76%
**Nvi4**				75%	62%	63%	57%	65%	60%	66%	66%	62%
**Nvi5**					63%	63%	55%	62%	62%	61%	62%	64%
**Nvi6**						60%	52%	60%	65%	59%	59%	62%
**Nvi7**							71%	74%	78%	82%	84%	75%
**Ace4**								66%	66%	73%	69%	66%
**Tca8**									70%	76%	73%	73%
**Tca9**										74%	76%	74%
**Phu5**											84%	77%
**Dpu6**												77%

* In the *T. spiralis* sequence only 5 TMs were used for comparisons.
