# Supplementary material for: Nematode and Arthropod Genomes Provide New Insights into the Evolution of Class 2 B1 GPCRs
Source: PLoS One. 2014 Mar 20;9(3):e92220. doi: 10.1371/journal.pone.0092220 (PMC3961327; doi:10.1371/journal.pone.0092220)
Supplement: Table S5 — Amino acid sequence similarity of the T. castaneum Class 2 B1 receptors. (PDF) [file pone.0092220.s010.pdf]

Table S5

	Tca2	Tca3	Tca4	Tca5	Tca6	Tca8	Tca9
**Tca1**	74%	62%	66%	51%	39%	61%	59%
**Tca2**		63%	63%	56%	37%	60%	59%
**Tca3**			74%	52%	41%	60%	64%
**Tca4**				56%	44%	64%	65%
**Tca5**					41%	51%	50%
**Tca6**						38%	40%
**Tca8**							70%
