# Supplementary material for: Nematode and Arthropod Genomes Provide New Insights into the Evolution of Class 2 B1 GPCRs
Source: PLoS One. 2014 Mar 20;9(3):e92220. doi: 10.1371/journal.pone.0092220 (PMC3961327; doi:10.1371/journal.pone.0092220)
Supplement: Table S6 — Percentages of amino acid sequence similarity of the C. elegans and T. castaneum receptors with the human homologues and sea urchin ( Strongylocentroyus purpuratus ) PDF-R-like. The T. castaneum cluster A member (Tca6) shares the lowest sequence similarity with the human receptors. (PDF) [file pone.0092220.s011.pdf]

Table S6

	C. elegans			*T. castaneum*							
Homo sapiens	***PDF-R***	***Seb-2***	***Seb-3***	***Tca1***	***Tca2***	***Tca3***	***Tca4***	***Tca5***	***Tca6***	***Tca8***	***Tca9***
Calcitonin subfamily											
CALCR	62%	55%	56%	60%	60%	65%	72%	57%	44%	59%	63%
CALCRL	59%	50%	55%	60%	60%	66%	72%	52%	44%	58%	61%
Corticotropin subfamily											
CRHR1	54%	50%	61%	64%	61%	60%	61%	51%	42%	65%	66%
Parathyroid subfamily											
PTHR1	58%	52%	55%	60%	60%	63%	63%	55%	42%	62%	69%
Glucagon subfamily											
GCGR	55%	53%	58%	59%	58%	62%	66%	51%	43%	65%	67%
GIPR	53%	54%	53%	59%	57%	61%	64%	53%	39%	64%	68%
Secretin subfamily											
VIPR1	57%	50%	59%	60%	60%	62%	65%	54%	42%	62%	65%
ADCYAP1R1	53%	50%	59%	62%	60%	59%	65%	52%	41%	65%	64%
Strongylocentrotus purpuratus											
PDF-R-like	60%	48%	54%	56%	50%	57%	58%	60%	46%	57%	60%
