# Supplementary material for: Nematode and Arthropod Genomes Provide New Insights into the Evolution of Class 2 B1 GPCRs
Source: PLoS One. 2014 Mar 20;9(3):e92220. doi: 10.1371/journal.pone.0092220 (PMC3961327; doi:10.1371/journal.pone.0092220)
Supplement: Table S7 — Gene list (accession number, chromosome position, symbol and initial gene position) of the nematode ( C. elegans ) and vertebrate human ( H. sapiens ) and chicken ( G. gallus ) gene sequence homologues of the T. castaneum Class 2 B1 gene environment on LG2, LG4, LG5 and LG9. Data was obtained using the Ensembl Biomart software. (PDF) [file pone.0092220.s012.pdf]

Table S7

T. castaneum				*C. elegans*				Human				Chicken			
Gene	Chr	Symbol	initial position (bp)	Gene	Chr	Symbol	initial position (bp)	Gene	Chr	Symbol	initial position (bp)	Gene	Chr	Symbol	initial position (bp)
TC000267	LG2	wcd	17565884	B0280.9	III		7120904	ENSG00000011260	17	UTP18	49337889	ENSGALG00000002985	18	UTP18	5070324
TC000391	LG2		14784411	ZK328.7	III		6032696	ENSG00000168026	3	TTC21A	39149152	ENSGALG00000010956	7	TTC21A	19154460
TC001148	LG2	mRpL32	14477869	C30C11.1	III	mrpl-32	8449757	ENSG00000106591	7	MRPL32	42971799	ENSGALG00000012337	2	MRPL32	51752758
TC000412	LG2	Abi	14425395	B0336.6	III	abi-1	5690107	ENSG00000108798	17	ABI3	47287589	ENSGALG00000001287	27	ABI3	3418377
TC000412	LG2		14425395	B0336.6	III	abi-1	5690107	ENSG00000138443	2	ABI2	204192942	ENSGALG00000008714	7	ABI2	13103070
TC000461	LG2		13639800	R13A5.9	III		7575275	ENSG00000151690	2	MFSD6	191273081	ENSGALG00000002280	7	MFSD6	98959
TC007472	LG4	lsn	4820988	C27F2.5	III	VPS-22	4960335	ENSG00000159210	17	SNF8	47006678	ENSGALG00000001309	27	SNF8	3476051
TC007279	LG4		7415248	T19D7.4	X		658521	ENSG00000198863	17	RUNDC1	41132582	ENSGALG00000002856	27	RUNDC1	5095037
TC007393	LG4		5900012	F25B5.3	III		5948890	ENSG00000141698	17	NT5C3B	39981335	ENSGALG00000003651	27	NT5C3B	4685681
TC008107	LG4		5860116	F35G12.4	III		4578855	ENSG00000114742	3	WDR48	39093489	ENSGALG00000006052	2	WDR48	5265323
TC013782	LG5	Aats-gly	5360724	T10F2.1	III	gars-1	5159729	ENSG00000106105	7	GARS	30634297	ENSGALG00000005694	2	GARS	4337075
TC014421	LG5		12662398	T12A2.15	III		6224430	ENSG00000117868	7		158523686				
TC014420	LG5		12658655	T12A2.15	III		6224430	ENSG00000117868	7		158523686				
TC014419	LG5	Esyt2	12654428	T12A2.15	III	esyt-2	6224430	ENSG00000117868	7	ESYT2	158523686	ENSGALG00000006542	2	ESYT2	9467167
TC014336	LG5		11147750	C26E6.12	III		4930600	ENSG00000105793	7	GTPBP10	89964537	ENSGALG00000009035	2	GTPBP10	21600607
TC014310	LG5	set2	10786564	K09F5.5	X	set-12	7733339	ENSG00000181555	3	SETD2	47057919	ENSGALG00000005508	2	SETD2	3873253
TC014286	LG5	Rpn3	10302884	C30C11.2	III	rpn-3	8447351	ENSG00000108344	17	PSMD3	38137050	ENSGALG00000022293	27	PSMD3	4314507
TC013701	LG5		7142432	F54F2.2	III	zfp-1	8792847	ENSG00000108292	17	MLLT6	36861795	ENSGALG00000001574	27	MLLT6	3971094
TC013743	LG5	Rcd-1	6101873	C26E6.3	III	ntl-9	4944513	ENSG00000144580	2	RQCD1	219433303	ENSGALG00000011402	7	RQCD1	21991654
TC013744	LG5	Imp	6053008	M88.5	III		4553646	ENSG00000136231	7	IGF2BP3	23349828	ENSGALG00000010961	2	IGF2BP3	31286666
TC013744	LG5		6053008	M88.5	III		4553646	ENSG00000159217	17	IGF2BP1	47074774	ENSGALG00000001293	27	IGF2BP1	3433057
TC011626	LG9	REG	21005961	Y66D12A.9	III		11568135	ENSG00000131467	17	PSME3	40976402	ENSGALG00000002937	27	PSME3	5060694
TC011738	LG9	Cdk12	18931833	B0285.1	III	cdtl-7	4333396	ENSG00000167258	17	CDK12	37617764	ENSGALG00000001731	27	CDK12	4158784
TC011797	LG9	Rab26	18146499	W01H2.3	X	rab-37	4227857	ENSG00000172794	17	RAB37	72666717	ENSGALG00000027789	18	RAB37	10703909
