# Supplementary material for: Nematode and Arthropod Genomes Provide New Insights into the Evolution of Class 2 B1 GPCRs
Source: PLoS One. 2014 Mar 20;9(3):e92220. doi: 10.1371/journal.pone.0092220 (PMC3961327; doi:10.1371/journal.pone.0092220)
Supplement: Table S8 — EST list and their tissue origin for the arthropod cluster A and cluster B Class 2 B1 receptor genes. Searches were performed using the deduced amino acid sequence of the species-specific genes identified in the study and their identity confirmed against the species genomes. (PDF) [file pone.0092220.s013.pdf]

Table S8

	Organism	Accession number	Tissue origin
**Cluster A**	*T. castaneum*	*DT770078.1, DT772679.1, DT773332.1, DT779360.1,DT779467.1, DT779872.1, DT782052.1*	Hindgut and malpighian tubules
		DT788191.1, DT796861.1	Whole larvae
	B.mori	*FS832340.1, FS833089.1,*	Mid-gut
		FS791659.1, FS791666.1	Fatbody
		FS728582.1, FS739156.1	Malpighian tubule
		CK529712.1, AV402071.1	Hemocyte
**Cluster B**	*T. castaneum*	*DT780536.1, EB752028.1*	Hindgut and malpighian tubules
		DN648669.1, DN647790.1	Mixed larvae or adults
	I. scapularis	EW907959.1, EW907960.1	Whole body
	A. melifera	DB754882.1, DB753685.1	Head
		BI507423.1, BI507803.1	Brain
		HX305996.1	Whole body
